# Engineered Bacteria Factory Integrating Drug Delivery and Antibody Manufacture for Activating the STING Signal Pathway Mediated Tumor Immunotherapy

**DOI:** 10.1002/advs.202518172

**Published:** 2026-02-19

**Authors:** Peng‐Shuo Dong, Tian Yao, Yong Li, Zi‐Hui Yan, Xiao‐Ting Xie, Jia‐Hua Zou, Kai Cheng, Jin‐Xuan Fan, Yuan‐Di Zhao, Qiu‐Ran Xu

**Affiliations:** ^1^ Britton Chance Center for Biomedical Photonics at Wuhan National Laboratory for Optoelectronics – Hubei Bioinformatics & Molecular Imaging Key Laboratory Department of Biomedical Engineering College of Life Science and Technology Huazhong University of Science and Technology Wuhan Hubei P. R. China; ^2^ Department of Oncology Huanggang Central Hospital of Yangtze University Huanggang Hubei P.R. China; ^3^ Hubei Clinical Medical Research Center of Esophageal and Gastric Malignancy Huanggang Hubei P.R. China; ^4^ Department of Chemistry The Chinese University of Hong Kong Hong Kong SAR P.R. China; ^5^ Zhejiang Key Laboratory of Tumor Molecular Diagnosis and Individualized Medicine Zhejiang Provincial People's Hospital Affiliated People's Hospital Hangzhou Medical College Hangzhou Zhejiang P. R. China

**Keywords:** engineered bacteria, immune checkpoint inhibitors, STING signal pathway, tumor immunotherapy

## Abstract

Macrophages show great potential for application in cellular immunotherapy, but are limited by immune checkpoints (ICBs). Immune checkpoint inhibitors (ICIs) can effectively block immune escape pathways and alleviate immune suppression in the tumor microenvironment (TME). Here, we constructed hypoxia response bacteria (HRB) that specifically expressed CD47 antibody (aCD47) under hypoxic conditions in the TME, enabling the in situ synthesis of ICIs at the tumor site. In addition, this study further prepared responsive liposomes for encapsulating the STING agonist cGAMP and modified them by covalent attachment to the HRB surface to form the composite material HRB@LC. This composite system can synergistically block CD47‐SIRPα‐mediated immune escape and activate the STING signal pathway, thereby enhancing systemic antitumor immune responses and significantly improving the efficacy of immunotherapy.

## Graphical abstract

To address the limitations of protein‐based ICIs in applications, this study constructed an engineered bacterium, HRB, capable of expressing aCD47 in hypoxic TME. Through the Schiff base reaction, responsive liposomes (LC) loaded with the STING agonist cGAMP were connected to the surface of bacteria to form HRB@LC. HRB@LC enhances systemic immune responses by synergistically blocking immune escape and activating the STING signal pathway, significantly inhibiting tumor growth.

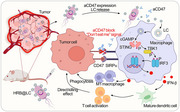

## 1 Introduction

Stimulator of interferon genes (STING) is an endoplasmic reticulum‐resident protein [1], which plays a central role in DNA‐triggered innate immune responses [2], and is critical for the expression of type I interferons [3, 4]. STING agonists have been widely studied in recent years for their ability to activate the STING signal pathway in anti‐tumor therapy. This triggers a local innate immune response in the tumor, effectively altering the tumor's immunosuppressive microenvironment. Under the effect of STING agonists, immune cells, including macrophages and dendritic cells (DCs) are induced to express type I interferons (IFN), which connect innate immune and adaptive immune responses by promoting antigen presentation [5, 6], polarizing macrophages [7, 8, 9], and maturing DCs [10, 11], further exert antitumor effects. Among them, macrophages, as an important component of innate immunity, have the characteristics of rapid immune response, independence from tumor antigens, and direct killing effects independent of T cells in antitumor therapy. They are considered one of the most promising immune cell therapies [12, 13, 14]. However, the CD47 molecule, which is overexpressed in various common tumor cells (including breast cancer, gastric cancer, and colorectal cancer) [15], can bind to the inhibitory receptor Signal Regulatory Protein alpha (SIRPα) on the surface of macrophages, transmitting a “don't eat me” signal, thereby inhibiting the phagocytic function of macrophages and mediating tumor immune escape [16, 17]. These macrophage immune checkpoint inhibitors (ICIs), represented by CD47 antibodies (aCD47), competitively bind to block tumor immune escape pathways, thereby alleviating immunosuppression [18, 19]. Existing ICIs are mainly protein‐based drugs represented by monoclonal antibodies. They have significant limitations during administration, including systemic toxicity due to off‐target effects, poor efficacy due to degradation, and unfavorable pharmacokinetic properties [20, 21]. Direct delivery of ICIs using nanocarriers [22, 23, 24], or in situ synthesis within cells by transfecting the mRNA sequence of ICIs can effectively address the above limitations [25, 26]. However, current nanocarriers are affected by the physical barriers and heterogeneity of the tumor extracellular matrix, resulting in uneven distribution of drugs within tumors. mRNA molecules have the disadvantages of poor stability and easy degradation, making it difficult to achieve continuous and stable in situ synthesis of ICIs. Therefore, there is an urgent need to develop novel delivery strategies for ICIs‐based therapies that combine precise targeting and in situ synthesis in the development of tumor immunotherapy.

Bacteria have efficient metabolic systems, various physiological functions, and simple cell structures. In recent years, they have been widely used as delivery platforms in cancer treatment [27, 28]. Many facultative anaerobic bacteria exhibit hypoxic tropism and can accumulate in the hypoxic tissues deep within solid tumors through their autonomous motility. Meanwhile, the nutrient‐rich and immunosuppressive tumor microenvironment (TME) provides favorable conditions for their colonization and proliferation. The rapid development of synthetic biology and nanotechnology has also provided powerful tools for the controlled engineering of bacteria. Artificially regulating the structure, function, and behavior of bacteria enables them to precisely synthesize active biomolecules, including proteins. This makes them flexible and efficient “living factories” for the in situ synthesis of ICIs [29, 30]. Therefore, using synthetic biology methods to construct engineered bacteria and nanotechnology to optimize their performance can effectively enable the controlled, sustained, in situ synthesis of ICIs at tumor sites [31, 32, 33]. This strategy is expected to effectively block tumor immune escape pathways and restore immune system function.

**FIGURE 1:**
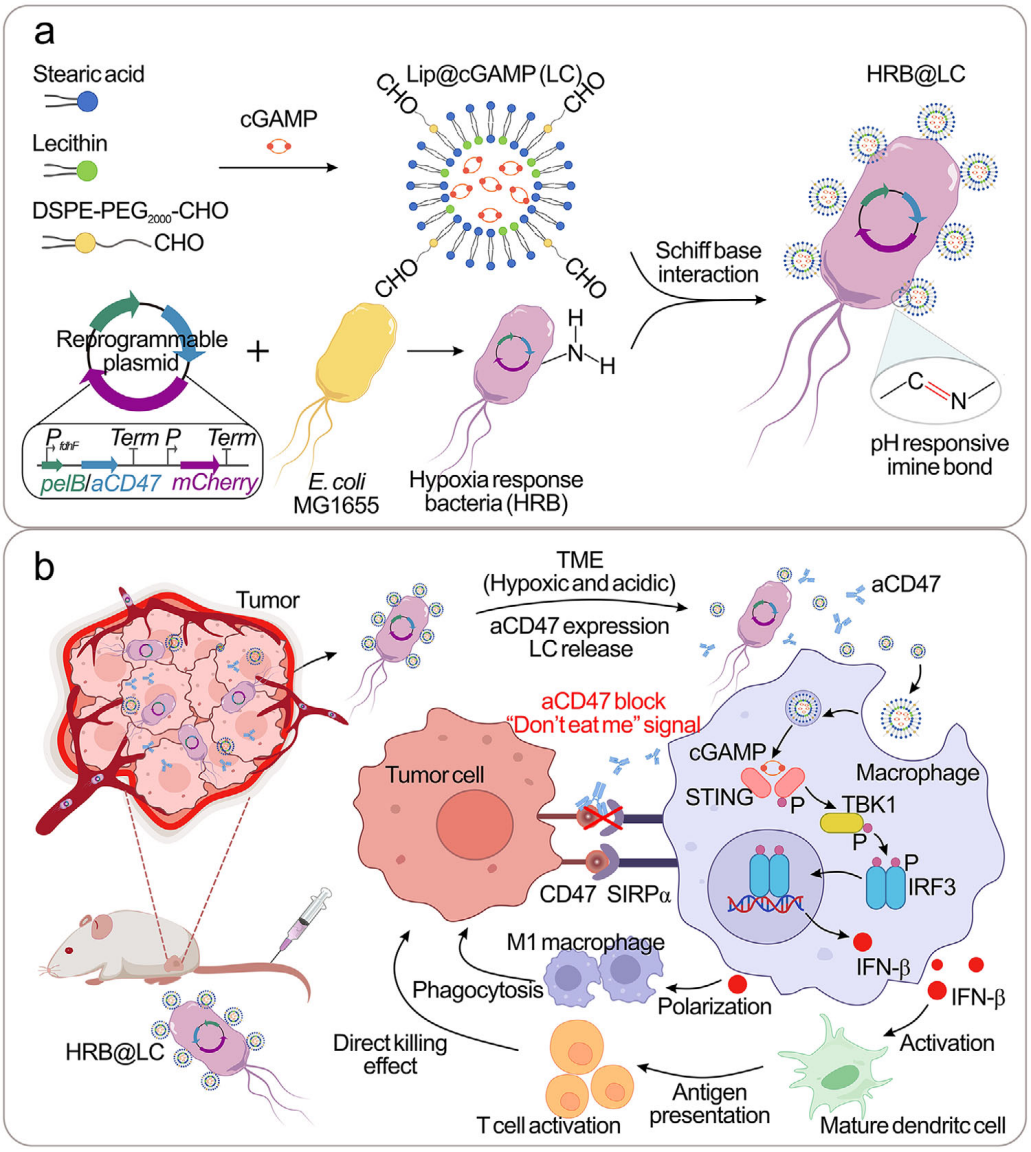
Synthesis and antitumor mechanism of HRB@LC. (a) Schematic diagram of HRB@LC preparation process. (b) Schematic diagram of the combination of engineered bacteria expressing ICIs and cGAMP‐loaded liposomes for drug delivery to tumor regions, enhancing anti‐tumor immunotherapy.

Studies have shown that the combination of ICIs and STING agonists can synergistically enhance the anti‐tumor immune response [34, 35]. Here, we reprogram bacteria through synthetic biology modification and material modification, enabling them to block tumor immune escape and activate the STING signal pathway simultaneously, thereby synergistically enhancing the efficacy of immunotherapy. Specifically, this study constructed hypoxia response bacteria (HRB) by transforming a reprogrammable plasmid containing the hypoxic promoter fdhF (*PfdhF*) and the pelB‐CD47 antibody (*aCD47*) sequence into attenuated *E. coli* MG1655, enabling the expression of aCD47 in the hypoxic TME. At the same time, we prepared responsive liposomes loaded with the STING agonist cGAMP (LC). Its hydrophilic core encapsulates cGAMP, and its surface is modified with aldehyde groups. Based on the Schiff base reaction [36], the aldehyde group on the LC surface covalently bonds with the amino group on the HRB surface to form HRB@LC. After targeting the tumor region, HRB responds to hypoxic TME by expressing aCD47, blocking the “don't eat me” signal mediated by CD47/SIRPα, and restoring the phagocytic function of macrophages. At the same time, LC responds to weakly acidic TME released from the HRB surface and is taken up by macrophages, activating the STING signal pathway, and inducing IFN‐β expression, which in turn triggers macrophage M1 polarization, T cell infiltration and activation, and DCs maturation (Figure 1). HRB@LC enhances systemic immune responses by synergistically blocking immune escape and activating the STING signal pathway, significantly inhibiting tumor growth. Simultaneously targeting both macrophages and T cells within the same therapeutic framework can synergistically engage innate and adaptive immune responses. This strategy combines the direct cytotoxic activity of T cells with the phagocytic function of macrophages, offering distinct advantages in anti‐tumor therapy, such as rapid immune activation and antigen‐independent responses. This study shows an innovative strategy for reprogramming bacteria through synthetic biology and materials engineering, providing a new paradigm for enhancing the efficacy of immunotherapy.

## 2 Results and Discussion

### 2.1 Preparation and Characterization of HRB@LC

**FIGURE 2:**
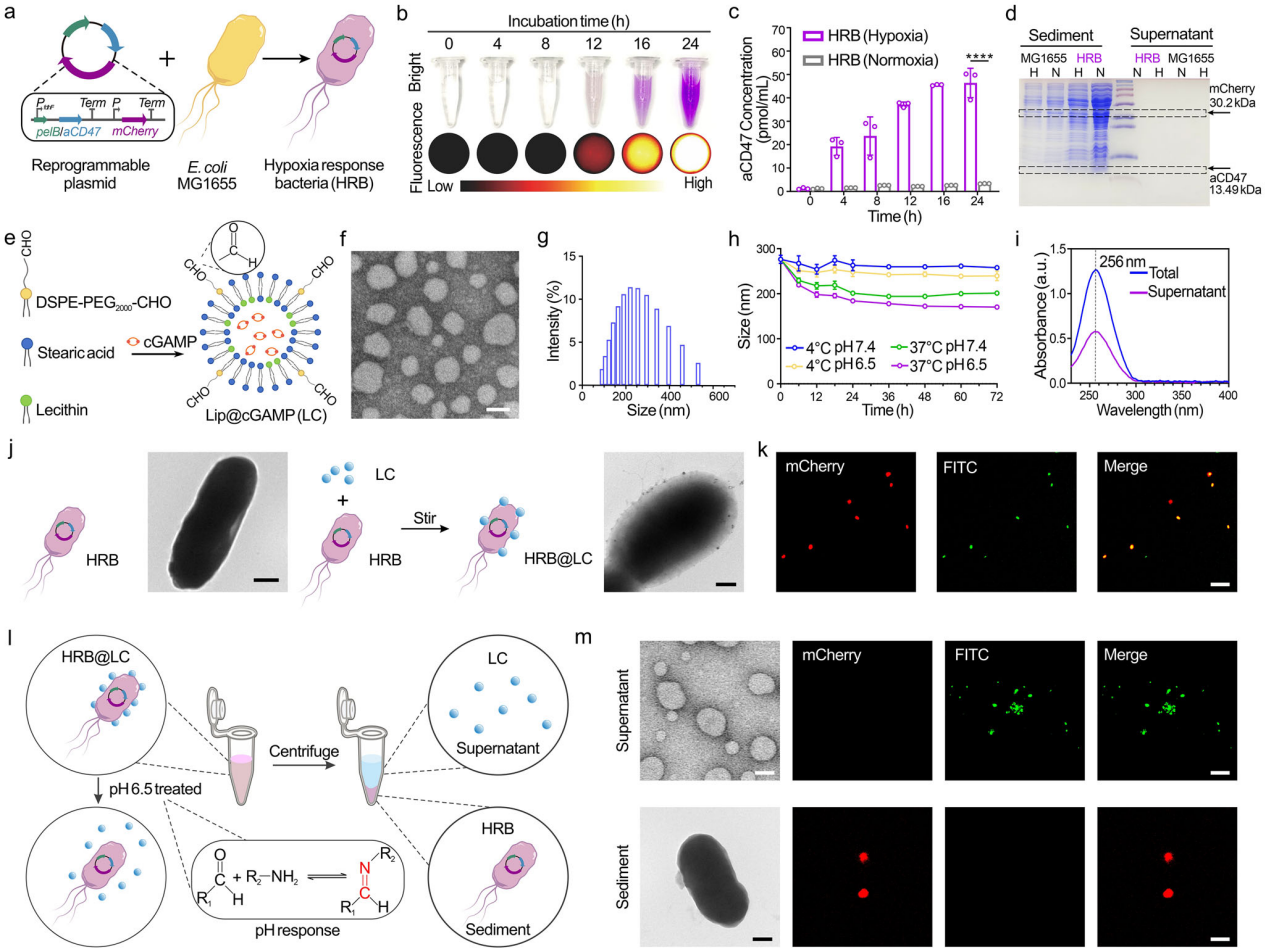
Preparation and Characterization of HRB@LC. (a) Schematic diagram of HRB construction process. (b) Visualisation and fluorescence images of mCherry expressed by HRB in a hypoxic environment. Images were representative of three experiments. h represents hour. (c) Quantitative analysis of aCD47 protein content in HRB supernatant. Data are presented as the means ± SD (n = 3). (d) Representative SDS‐PAGE image of aCD47 and mCherry proteins expressed in HRB and MG1655. Images were representative of three experients. N represents normoxia, and H represents hypoxia. (e) Schematic diagram of LC preparation process. (f) TEM image of LC. Scale bar, 50 nm. (g) Hydrated particle size distribution graph of LC. (h) Hydrated particle size of LC under different temperature and pH environments. Data are presented as the means ± SD (n = 3). (i) UV/Vis absorbance spectra of LC before and after ultrafiltration. Total represents the cGAMP solution before ultrafiltration, and Supernatant represents the filtrate obtained after ultrafiltration. (j) Schematic diagram of HRB@LC preparation process. TEM images of HRB and HRB@LC. Scale bars, 250 nm (HRB) and 300 nm (HRB@LC). (k) Confocal microscopy images of HRB@LC. Images were representative of three experiments. Scale bar, 20 µm. (l) Schematic diagram of the imine bond in HRB@LC breaking in response to a weak acidic environment. (m) TEM images and confocal microscopy images of the supernatant and sediment after treating HRB@LC in a weak acid environment. Scale bars, 50 nm (TEM image of the supernatant), 250 nm (TEM image of the sediment), and 10 µm (confocal microscopy images). ^*^: *P* < 0.05. ^**^ *P* < 0.01. ^***^: *P* < 0.001.

We constructed a prokaryotic expression plasmid containing the anaerobic promoter *fdhF*. This reprogrammable plasmid includes the *aCD47* gene with the pelB secretion signal peptide at the N‐terminus and the independently expressed *mCherry* tracer gene. By transforming the reprogrammable plasmid into *E. coli* MG1655 using the heat shock method, we can obtain HRB that stably expresses the reprogrammable plasmid (Figure 2a). To verify the stable expression of the reprogrammable plasmid in HRB, we detected HRB under hypoxic conditions at different time points using visual imaging and fluorescence imaging. As shown in Figure 2b, as the culture time increases, the bacterial suspension gradually turns purple, and the fluorescence intensity also gradually increases. This is due to the expression of mCherry by HRB. The supernatant of HRB was collected for ELISA detection, and the results are shown in Figure 2c. Under hypoxic conditions, the content of aCD47 in the supernatant of HRB bacterial culture reached 50 pmol/mL with increasing culture time, while under normoxia conditions, the content of aCD47 was very low. Consistent with the ELISA results, SDS‐PAGE experiments showed (Figure 2d) that under hypoxic conditions, the supernatant and bacterial lysate of HRB contained a large amount of aCD47, while under normoxic conditions, it was almost undetectable. In addition, the mCherry protein can be normally expressed under both normoxic and hypoxic conditions. In contrast, there is no expression of aCD47 and mCherry in the control group MG1655. The above results prove the successful transformation and stable expression of the reprogrammable plasmid in HRB, meaning that HRB can express and release aCD47 under hypoxic conditions, while HRB can express mCherry as a tracer.

Lip@cGAMP (LC), a responsive liposome encapsulating the STING agonist cGAMP, was prepared using the thin film hydration method. LC's hydrophilic core encapsulates cGAMP, and its surface is modified with aldehyde groups (‐CHO) (Figure 2e). Transmission electron microscopy (TEM) imaging result (Figure 2f) shows that LC is uniformly dispersed in spherical form, with a size of 50–100 nm. In addition, the hydrated particle size of LC is 218 nm, and the polydispersity index (PDI) is 0.331 (Figure 2g). These results indicate that LC is a spherical particle with a uniform size distribution and a suitable size for loading on the surface of bacteria. To further verify the stability of LC, we monitored changes in the hydrated particle size of LC under different temperature and pH conditions. The results were shown in Figure 2h, where the hydrated particle size of LC remained essentially stable over 72 h. Specifically, there was a certain degree of decrease in particle size within the first 12 h, followed by stabilization. The UV/Vis absorbance spectra results (Figure 2i) show that cGAMP exhibits a characteristic absorption peak at 256 nm, and the quantitative calculation of the LC encapsulation rate is 10.08%. The drug loading capacity of LC was calculated to be 0.051%.

The aldehyde group on the surface of LC reacts with the amino group on the surface of HRB through a Schiff base reaction to form an imine bond, connecting LC to the surface of HRB to form HRB@LC. TEM imaging result (Figure 2j) shows that the untreated HRB surface is smooth, while after successful loading of LC, the spherical particles can be clearly observed adhering to the surface of HRB. To further demonstrate the successful synthesis of HRB@LC, we added 1% DSPE‐FITC into the LC preparation process for fluorescent labeling. Confocal microscopy imaging revealed perfect colocalization between the mCherry fluorescence carried by HRB and the FITC fluorescence carried by LC (Figure 2k). Additionally, fluorescence colocalization analysis was performed, yielding a colocalization scatter plot (Figure S1). The diagonal distribution of a significant population of pixels indicates a high degree of colocalization, suggesting the construction of HRB@LC. The analysis revealed a Pearson's coefficient of 0.74 (a value greater than 0.5 indicates colocalization), suggesting strong colocalization between HRB and LC. Correspondingly, we prepared a liposome without ‐CHO groups using DSPE‐PEG2000. These liposomes were similarly FITC‐labeled. After overnight mixing with HRB, confocal microscopy and TEM imaging revealed (Figure S2) that the liposome without CHO groups could not load onto the HRB surface. The above results indicate that LC successfully covalently bonded to the HRB surface through the Schiff base reaction, forming HRB@LC. We further evaluated the stability of LC on HRB@LC by fluorescence imaging. As shown in Figure S3, the amount of LC‐FITC present in HRB@LC after settling for 48 h showed no significant difference compared with that in freshly prepared HRB@LC. However, the imine bond formed by the Schiff base reaction will break down in an acidic environment. We verified the acid‐responsive breakdown characteristics of its covalent bonding by treating HRB@LC in a weak acidic environment (Figure 2l). The HRB@LC solution treated with weak acid was centrifuged, and the supernatant and precipitate were collected and subjected to TEM and confocal microscopy imaging, respectively. The results are shown in Figure 2m. The supernatant contains released LC: TEM image shows that LC remains uniformly spherical, with a size of 50–100 nm; confocal microscopy images show that only the FITC fluorescence carried by LC is present in the supernatant, with no mCherry fluorescence carried by HRB. The sediment contained HRB without LC: TEM image showed that the HRB surface was smooth with no spherical particles attached, and confocal microscopy images showed that the precipitate contained only mCherry fluorescence carried by HRB, with no FITC fluorescence carried by LC. These results indicate that LC responds to weakly acidic environments and can successfully release from the HRB surface. In addition, the hydrated particle size of LC in the supernatant was 200 nm, with a PDI of 0.280 (Figure S4), indicating that weak acid treatment did not destroy the morphology and size of LC.

### 2.2 Penetration Capability and Tumor Targeting of HRB@LC

**FIGURE 3:**
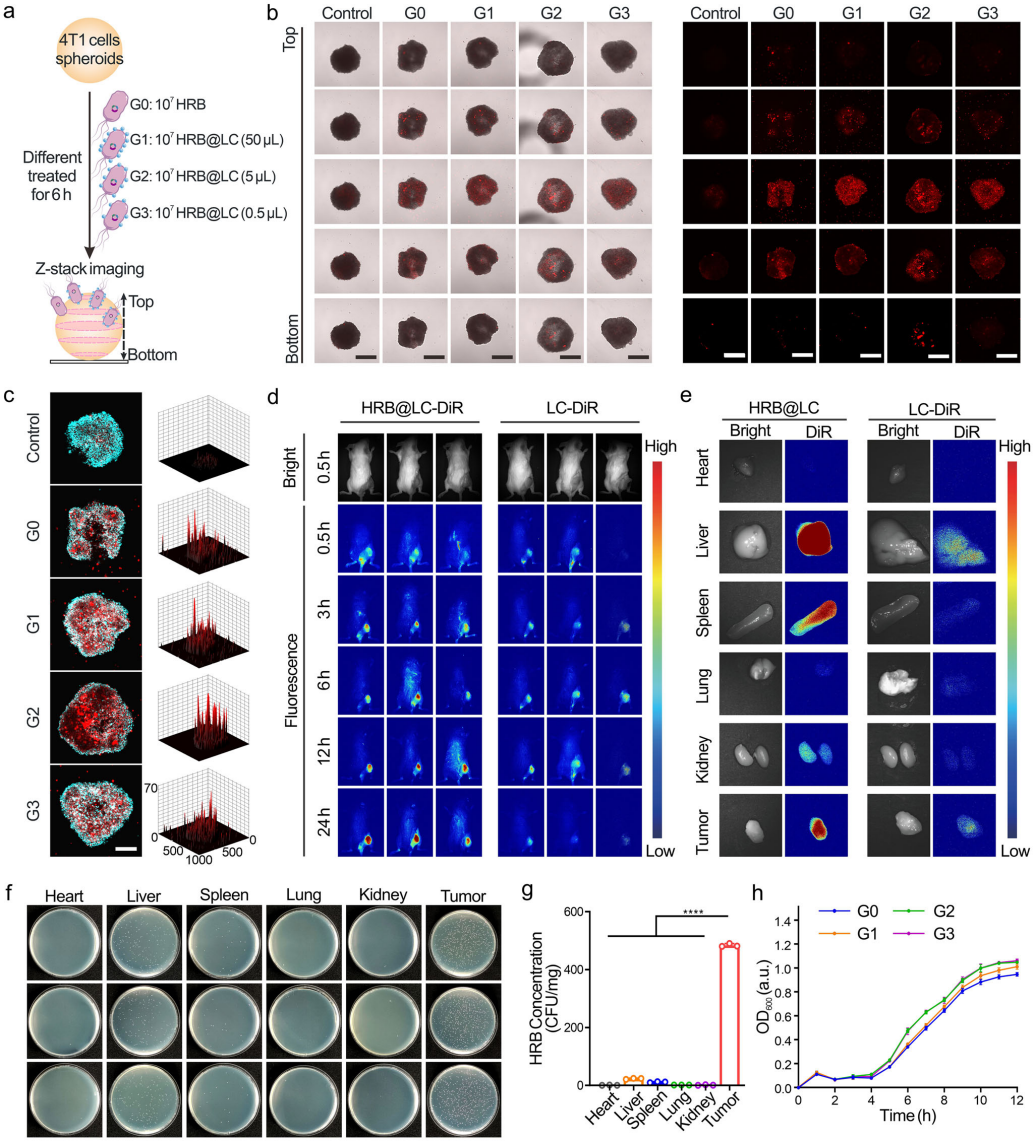
HRB@LC tumor targeting and penetrating capacity. (a) Schematic diagram of the penetration experiments of HRB@LC and HRB in 4T1 cell spheroids. (b) Penetration of HRB@LC and HRB in 4T1 cell spheroids. Images were representative of three experiments. Scale bars, 400 µm. (c) Confocal microscopy images of the permeation about HRB@LC and HRB in the central position of 4T1 cell spheroids and mCherry intensity surface plot. Blue fluorescence represents Hoechst 33342‐stained 4T1 cell spheroids. Scale bar, 200 µm. (d) Fluorescence images of tumor‐bearing mice with different treatments at different time points. n = 3. (e) Fluorescence images of major organs and tumor tissues of tumor‐bearing mice. Images were representative of three experiments. (f) Homogenates of major organs and tumor tissues of mice after injection with HRB@LC on 24 h, then cultured on solid LB agar plates and photographed. (g) Numbers of bacteria (HRB) were quantified by plate counting. Data are presented as the means ± SD (n = 3). (h) Bacterial growth curves of HRB and HRB@LC. Data are presented as the means ± SD (n = 3). The meanings represented by G0‐G3 are consistent with Figure 3a. ^*^: *P* < 0.05. ^**^ *P* < 0.01. ^***^: *P* < 0.001.

To assess the effect of LC loading on HRB penetration capability, we prepared HRB@LC with different LC loadings by setting a connection ratio gradient and investigated its penetration capability in 4T1 cells spheroids (Figure 3a). The results of the Z‐axis scan are shown in Figure 3b. The HRB loaded with LC (G1‐G3) and the HRB without LC (G0) both show gradually increasing mCherry fluorescence at the center of the 4T1 cells spheroids. The fluorescence intensity analysis in Figure 3c further demonstrates the deep penetration of HRB and HRB@LC into 4T1 cell spheroids. These results indicate that the composite material HRB@LC maintains the Penetration capability of HRB while efficiently loading LC, which is crucial for HRB to exert its antitumor effect.

DiR was added during the LC preparation process to label it, forming LC‐DiR to assess the tumor targeting ability and in vivo distribution of HRB@LC. The loading amount of LC in HRB@LC was determined by in vitro fluorescence imaging analysis (Figure S5). We injected HRB@LC‐DiR (10^7^ CFU) and an equal amount of LC‐DiR (5 µL) into tumor‐bearing mice via the tail vein and performed in vivo fluorescence imaging at 0.5, 3, 6, 12, and 24 h after injection. As shown in Figure 3d, HRB@LC‐DiR showed aggregation of red fluorescent signals at the tumor site as early as 3 h post‐injection, with the signals persisting up to 24 h. In contrast, LC‐DiR only showed weak fluorescence at the tumor site 6 h post‐injection, and the fluorescent signals had largely disappeared by 24 h. The above results indicate that HRB@LC has significant tumor targeting ability and can effectively deliver and retain the loaded LC in the tumor site. Although free LC can be passively targeted to tumor regions by enhanced permeability and retention effect (EPR), its accumulation and retention time at the tumor site are much lower than those of HRB@LC. We collected tumor tissues and major organs (heart, liver, spleen, lung, and kidney) from mice 24 h after injection for fluorescence imaging. The results (Figure 3e) were consistent with the in vivo imaging results. Tumor tissues showed the strongest red fluorescence, and the fluorescence intensity of HRB@LC was significantly higher than free LC. Next, we homogenized the collected mouse tumor tissues and major organs, spread them on solid LB agar plates containing ampicillin (50 µg/mL), cultured them at 37°C overnight, photographed them, and counted the number of HRB. As shown in Figure 3f,g, HRB exhibited the highest colonization levels in tumor tissues, maintaining approximately 500 CFU/mg even 24 h post‐injection. Only minute amounts of HRB were detected in the liver, and it was barely undetectable in other organs. This indicates that HRB@LC possesses significant tumor targeting capability, enabling it to selectively colonize tumor sites efficiently while exhibiting extremely limited distribution in normal organs. This is crucial for assessing its safety and efficacy. Finally, we also explored the effect of LC loading on HRB proliferation capacity. The growth curves of HRB and HRB@LC were continuously monitored. The results are shown in Figure 3h, All groups reached the logarithmic growth phase at around 6 h and reached the plateau phase at around 12 h, proving that LC loading does not affect HRB proliferation capacity. The concentration of aCD47 in the supernatant was measured using an ELISA kit after culturing HRB@LC and HRB under hypoxic conditions. As shown in Figure S6, both HRB@LC and HRB secreted approximately 20 pmol/mL of aCD47 after about 10 h of culture, demonstrating that LC loading does not impair the antibody expression capability of HRB.

### 2.3 HRB@LC Activates the STING Signal Pathway in Macrophages and Blocks the “CD47‐SIRPα” Signal

**FIGURE 4:**
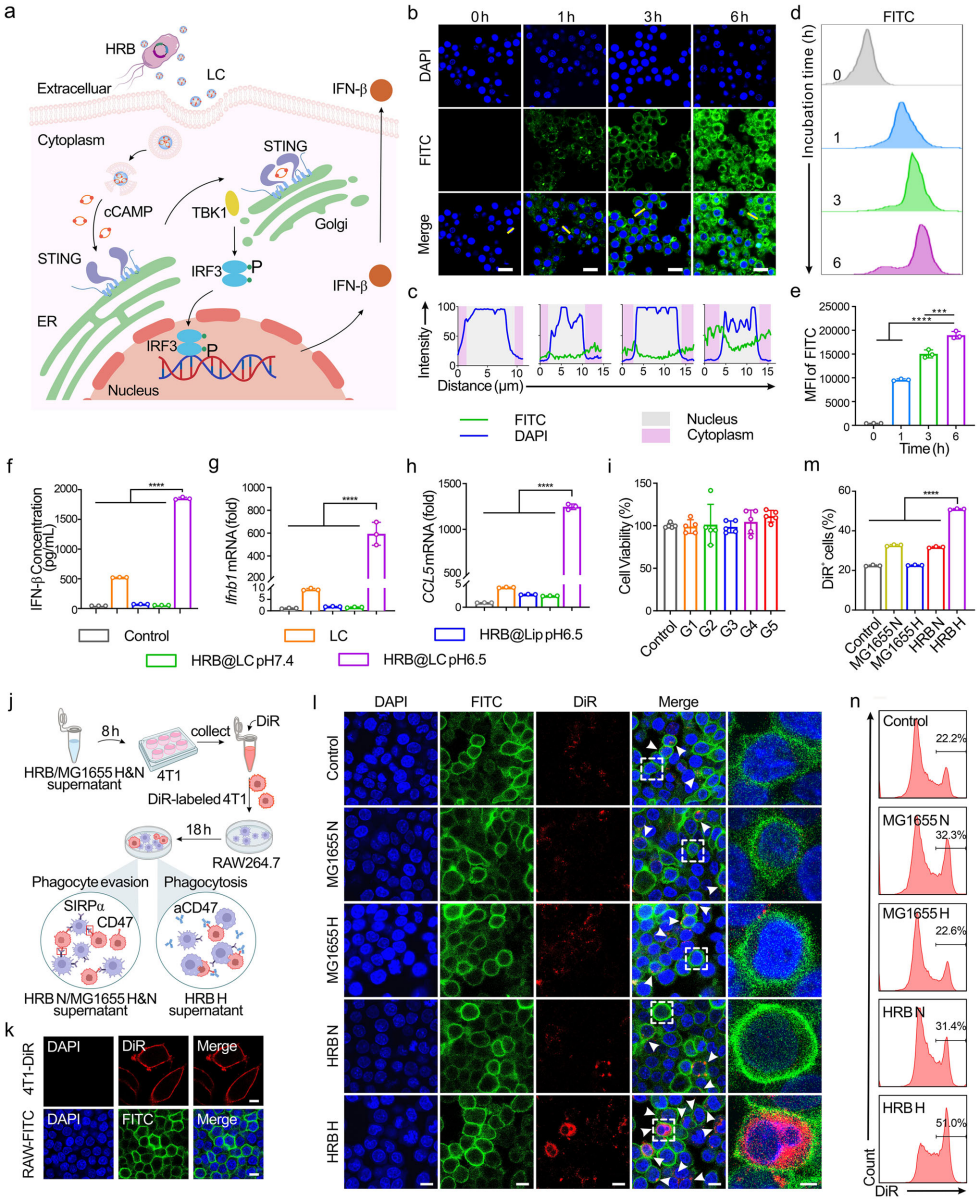
HRB@LC activates the STING signal pathway and blocks the “CD47‐SIRPα” signal. (a) Schematic diagram of the mechanism by which HRB@LC responds to an acidic environment by releasing LC, which is then phagocytosed by macrophages and ultimately activates the STING signal pathway. (b) Confocal microscopy images of macrophages RAW 264.7 cells phagocytose LC‐FITC. Blue represents the cell nucleus of RAW 264.7 cells, green represents LC, and yellow solid lines represent the cells selected for fluorescence quantitative analysis. Scale bars, 20 µm. Images were representative of three experiments. (c) Fluorescence quantitative analysis of macrophages RAW 264.7 cells phagocytose LC‐FITC. The green lines represent FITC, the blue lines represent DAPI, the gray areas represent the nucleus, and the purple areas represent cytoplasm. (d‐e) Representative flow cytometry (d) and quantitative analysis (e) of LC‐FITC in RAW 264.7 cells. Data are presented as the means ± SD (n = 3). (f) Quantitative analysis of IFN‐β protein content in the supernatant of RAW 264.7 cells after different treatments for 4 h. Data are presented as the means ± SD (n = 3). (g‐h) Quantification of relative gene expression about *Ifnb1* (g) and *CCL5* (h) in RAW 264.7 cells after different treatments for 4 h. Data are presented as the means ± SD (n = 3) (i) Cell viability of 4T1 cells after different treatments. Data are presented as the means ± SD (n = 5). G1 represents HRB@LC (10^3^ CFU@10^−4 ^µg), G2 represents HRB@LC (10^4^ CFU@10^−3^ µg), G3 represents HRB@LC (10^5^ CFU@10^−2^ µg), G4 represents HRB@LC (10^6^ CFU@10^−1^ µg), G5 represents HRB@LC (10^7^ CFU@1 µg). (j) Schematic diagram of co‐incubation experiments between 4T1 cells and RAW 264.7 cells. (k) Confocal microscopy images of DiR‐labeled 4T1 cells and FITC‐labeled RAW 264.7 cells. Scale bars, 10 µm. (l) Confocal microscope images of 4T1 cells and RAW 264.7 cells co‐incubated after different treatments. Scale bars, 10 µm and 3 µm (enlarged view). The white arrows mark the phagocytosis, and the white dotted boxs mark the enlarged view area. Images were representative of three experiments. (m and n) Representative flow cytometry and quantitative analysis of DiR^+^ cells in RAW 264.7 cells. Images were representative of three experiments. Data are presented as the means ± SD (n = 3). ^*^: *P* < 0.05. ^**^ *P* < 0.01. ^***^: *P* < 0.001.

After HRB@LC targets the tumor sites, it responds to the acidic TME by releasing LC, which is then phagocytosed by macrophages and ultimately activates the STING signal pathway. (Figure 4a). Specifically, cGAMP binds specifically to STING, inducing a conformational change and activating it. Activated STING is then transported from the endoplasmic reticulum (ER) to the Golgi, where it recruits and activates TANK‐binding kinase 1 (TBK1). TBK1 is activated through autophosphorylation, which in turn phosphorylates interferon regulatory factor 3 (IRF3) and STING. Phosphorylated IRF3 forms dimers and translocates to the cell nucleus, ultimately inducing the expression of IFN‐β. To verify the phagocytosis of macrophages against LC, we added 1% DSPE‐FITC during the LC preparation process to obtain HRB@LC‐FITC. After treatment with a weak acid (simulating the TME), the supernatant was collected by centrifugation. The free LC‐FITC released from the HRB@LC‐FITC surface was obtained. They were then co‐incubated with RAW 264.7. cells. Cells were collected at different time points for confocal microscopy imaging and flow cytometry analysis. The results are shown in Figure 4b: After 1 h of incubation, the phagocytosis of RAW 264.7 cells was low; after 3 h of incubation, phagocytosis increased significantly, but the liposomes were mainly distributed in the cell membrane; after 6 h of incubation, the liposomes had been significantly internalized into the cytoplasm. To further quantify this process, we performed fluorescence intensity line scanning analysis on representative cells (marked with yellow solid lines). The results are shown in Figure 4c. At 6 h group, the FITC fluorescence intensity in the cytoplasm (purple area) was significantly higher than that in the 3 h group. Additionally, the flow cytometry results in Figure 4d,e are consistent with the confocal microscopy images, showing that the phagocytosis efficiency of RAW 264.7 cells toward liposomes increases with increased incubation time. These results indicate that LC responds to a weakly acidic environment by releasing from the HRB surface and being phagocytosed into the cytoplasm by macrophages.

To verify whether cGAMP released after LC internalization into the cytoplasm can effectively activate the STING signal pathway and induce IFN‐β expression, we designed the following experiments: HRB@LC was treated with a weak acid (simulating the TME) and centrifuged. The supernatant was added to DMEM and incubated with RAW 264.7 cells for 4 h. We set up three control groups: 1) HRB@LC untreated group (supernatant not treated with weak acid); 2) HRB@Lip treated with weak acid group (loaded empty liposomes); 3) Free LC group (equal dose). After incubation, collect the cell supernatant and use an IFN‐β ELISA assay kit to detect the IFN‐β content. At the same time, extract the total RNA from the cells and perform reverse transcription, then use real‐time quantitative polymerase chain reaction (RT‐qPCR) to detect the relative expression levels of the relevant genes. ELISA results showed (Figure 4f) that both the HRB@LC treated with the weak acid group and the free LC group could induce RAW 264.7 cells to express high levels of IFN‐β, and the former expresses more. There is no IFN‐β was detected in the other groups. RT‐qPCR results (Figure 4g) showed that HRB@LC treated with weak acid and free LC significantly increased the expression level of the *Ifnb1* gene (encoding IFN‐β), consistent with the ELISA experimental results. These data prove that LC responds to weak acidic environments by releasing from the HRB surface and being phagocytosed by macrophages, thereby activating the STING signal pathway in macrophages and promoting the expression of IFN‐β. Untreated HRB@LC cannot be phagocytosed due to the presence of imine bonds, preventing LC from being released and thus unable to activate the STING signal pathway. HRB@Lip treated with a weak acid also fails to activate the STING signal pathway because the liposomes do not contain cGAMP. The effect of free LC is lower than that of HRB@LC, which may be due to the pro‐inflammatory stimulation and influence of bacterial secretions on macrophages. It is worth noting that the expression level of the *CCL‐5* gene in the treated cells was also significantly increased (Figure 4h), suggesting that the activation of the STING signal pathway plays a role in T cell recruitment and activation. To more directly validate the activation of the STING pathway induced by LC, we performed Western blot analysis. Primary bone marrow‐derived macrophages (BMDM) were isolated from the bone marrow of 6‐week‐old BALB/c mice and then treated with LC for 10 h. Cells were collected for Western blot detection. As shown in Figure S7, LC treatment increased the intracellular levels of p‐IRF3 and p‐TBK1, which are signs of STING pathway activation. To prove the biological safety of the composite material HRB@LC, we conducted cell viability testing using the methylthiazolydiphenyl‐tetrazolium bromide (MTT) colorimetric method. The results are shown in Figure 4i, treatment with different concentrations of HRB@LC did not affect cell survival rates, demonstrating that HRB@LC has good safety.

Next, to see if the aCD47 expressed by HRB could successfully block the “CD47‐SIRPα” signal pathway, alleviate macrophage immune suppression, and boost their phagocytic ability, we did a co‐incubation experiment with 4T1 cells and mouse macrophages RAW 264.7 cells. The specific operations were as follows: HRB and MG1655 were cultured under normoxic or hypoxic conditions for 12 h, collect their supernatant. The supernatant was then co‐incubated with 4T1 cells for 8 h and labeled with DiR dye to mark the cell membrane. The labeled 4T1 cells were then co‐incubated with RAW 264.7 cells for 18 h, and the phagocytic effect was analyzed using confocal microscopy imaging and flow cytometry (Figure 4j). Figure 4k shows: FITC labels macrophage membranes, DAPI labels cell nuclei, and DiR labels 4T1 cell membranes. Figure 4l shows that 4T1 cells treated with hypoxic HRB supernatant exhibited a significant increase in phagocytosis events when co‐incubated with macrophages (white arrows indicate phagocytosed red 4T1 cells). The enlarged view clearly shows green macrophages enveloping red tumor cells, indicating that macrophages successfully phagocytosed 4T1 cells. The number of phagocytosis events in the other groups was significantly lower than that in the hypoxic HRB group. From a mechanistic perspective, the supernatant of hypoxic HRB contains a large amount of aCD47, which competitively binds to CD47 on the surface of 4T1 cells, blocking its interaction with SIRPα on macrophages. This blocks the “CD47‐SIRPα” signaling pathway and promotes the phagocytic function of macrophages. In contrast, in the immune escape groups, due to the absence of aCD47, CD47 on the surface of tumor cells can bind normally to SIRPα on the surface of macrophages, thereby inhibiting macrophage phagocytic activity and leading to immune escape of tumor cells. Therefore, no 4T1 was observed in the experimental results of these groups. Flow cytometry results and statistical analysis (Figure 4m,n; Figure S8) further revealed that the proportion of DiR^+^ cells in macrophages from the hypoxic HRB group was significantly higher than in other groups, indicating that hypoxic HRB group had the strongest phagocytic activity. In summary, hypoxia‐induced expression of aCD47 by HRB efficiently blocks the “CD47‐SIRPα” signal pathway, alleviates immunosuppressive effects, and enhances macrophage phagocytic capacity.

### 2.4 Inhibition of Tumor Growth and Activation of Antitumor Immune Responses by HRB@LC

**FIGURE 5:**
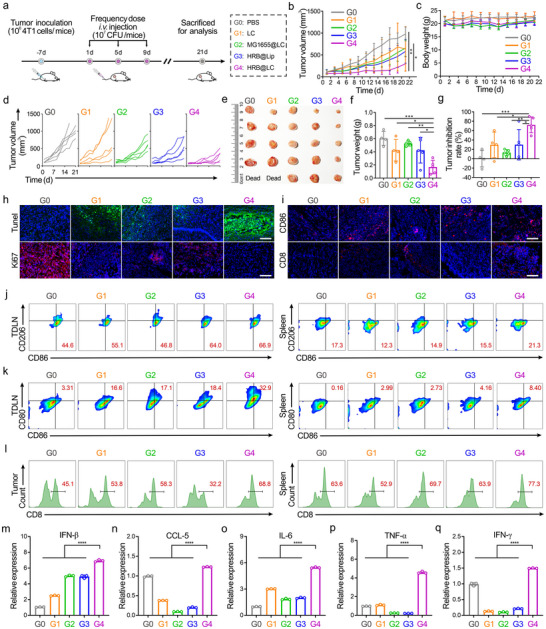
HRB@LC inhibits subcutaneous tumor growth and activates anti‐tumor immunity. (a) Schematic showing the treatment schedule in a subcutaneous breast cancer mouse model. d represents day. (b‐c) Tumor volume (b) and body weight (c) change curve of mice. Data are presented as the means ± SD (n = 5). (d) Tumor volume change curves of an individual mouse. (e‐g) Tumor images (e), tumor weights (f), and tumor suppression rate (g) obtained after different treatments. Data are presented as the means ± SD (n = 5). (h) Immunofluorescence staining (TUNEL and Ki‐67) of tumor tissues from mice after different treatments. Scale bars, 100 µm. (i) Immunofluorescence staining (CD86 and CD8) of tumor tissues from mice after different treatments. The red color above represents M1 macrophages, the red color below represents cytotoxic T cells, and the blue color represents the nucleus. Scale bars, 100 µm. (j) Representative flow cytometry of M1 macrophages (CD86^+^ CD206^−^) in TDLN and spleen from mice after different treatments. (k) Representative flow cytometry of mature DCs (CD80^+^ CD86^+^) in TDLN and spleen from mice after different treatments. (l) Representative flow cytometry of cytotoxic T cells (CD3^+^ CD8^+^) in tumor and spleen from mice after different treatments. Images were representative of three experiments. (m‐q) Quantification of relative gene expression about IFN‐β (m), CCL‐5 (n), IL‐6 (o), TNF‐α (p), and IFN‐γ (q) in tumor tissues from mice after different treatments. Data are presented as the means ± SD (n = 3). ^*^: *P* < 0.05. ^**^ *P* < 0.01. ^***^: *P* < 0.001.

To evaluate the in vivo antitumor effect of HRB@LC, we established a subcutaneous breast cancer mouse model and performed treatment according to the experimental scheme shown in Figure 5a. We randomly divided the tumor‐bearing mice into five groups, including the PBS group (G0), LC group (G1), MG1655@LC group (G2), HRB@Lip group (G3), HRB@LC group (G4), and gave them three doses, measuring tumor volume and mouse weight every other day. The results showed that the average tumor volume in the G4 group was the smallest compared to the other groups (Figure 5b). During the entire treatment period, there were no significant changes in body weight among the five groups of mice (Figure 5c). Additionally, continuous monitoring of tumor volume in each mouse showed that tumor growth was slowest in the G4 group (Figure 5d). On day 21, the tumors were collected for photography (Figure 5e) and weighing (Figure 5f). The tumors in the G4 group were the smallest and lightest, showing significant statistical differences compared to the other four groups. In addition, the average tumor inhibition rate in the G4 group reached 70% (Figure 5g), which was significantly higher than that in other groups. We also performed H&E staining on tumor tissues and major organs (heart, liver, spleen, lung, and kidney) of mice (Figure S9). The results showed that the G4 group had the least dense areas in tumor tissues, and no significant pathological changes were observed in the major organs of the five groups of mice. We further assessed the safety profile of HRB@LC in mice. Serum samples were collected on day 7 post‐injection, and six liver function parameters were measured. The results are presented in Figure S10. The results indicated that no significant differences were observed in the six liver function parameters between the HRB@LC and PBS groups. These data collectively prove that HRB@LC has excellent tumor suppression capabilities and biological safety. The survival rate of mice was 100% throughout the experimental period, with a tumor recurrence rate of 0%. Tumor tissues were analyzed using the TUNEL (terminal deoxynucleotidyl transferase dUTP terminal labeling) assay and Ki‐67 immunofluorescence staining, with results shown in Figure 5h. Compared to the control group, the G4 group exhibited significantly enhanced green fluorescence, indicating higher levels of tumor cell apoptosis in the G4 group; meanwhile, the red fluorescence in the G4 group exhibited a significant reduction, indicating that it significantly inhibited tumor proliferation. These results further confirm the efficacy of HRB@LC in inhibiting tumors.

Subsequently, we explored if HRB@LC activated the immune response within the tumor. We performed immunofluorescence staining analysis of tumor tissue sections for CD86 (a marker of M1 macrophages) and CD8 (a marker of cytotoxic T cells). As shown in Figure 5i, the G4 group exhibited the strongest CD86 and CD8 positive signals (red fluorescence), indicating the highest infiltration of M1 macrophages and CD8^+^ T cells in the tumor tissues of the G4 group. To further verify the activation and infiltration of immune cells in mice by HRB@LC, we used flow cytometry to detect infiltrating macrophages (Figure 5j; Figure S11), DCs (Figure 5k; Figure S12), and T cells (Figure 5l; Figures S13 and S14) in the TDLN, spleen, and subcutaneous tumors of mice. The results showed that, compared with the control group, G4 had the highest levels of M1 macrophages (CD86^+^ CD206^−^), mature DCs (CD80^+^ CD86^+^), and cytotoxic T cells (CD3^+^ CD8^+^) in TDLN, spleen, and subcutaneous tumors. These results indicate that HRB@LC effectively induces M1 polarization of macrophages, maturation of DCs, activation of T cells, and effective activation of antitumor immune responses in vivo. In addition, we performed quantitative analysis of the expression of inflammation‐related genes and STING signal pathway genes in tumor tissues through RT‐qPCR experiments. Figure 5m shows that, compared with the control group, the expression of IFN‐β, a downstream effector factor of the STING signal pathway, was significantly upregulated in G4, indicating that HRB@LC successfully activated the STING signal pathway. Figure 5n shows that the expression of CCL‐5, a cytokine used to recruit and activate T cells, was significantly higher in G4 than in the control group, suggesting that HRB@LC promoted the recruitment and activation of T cells within the tumor. Figure 5o,p shows that the expression of key immune stimulatory factors IL‐6 and TNF‐α in G4 was significantly higher than that in the control group. Given that IL‐6 and TNF‐α not only play an important role in antitumor effects but are also marker genes of M1 macrophages, their upregulation suggests polarization of macrophages toward the M1 like. More importantly, Figure 5q further shows that the expression of IFN‐γ, which can induce M1 polarization of macrophages, was also significantly increased in the G4 group. These data are consistent with the results of immunofluorescence staining and flow cytometry described above. Taken together, they indicate that HRB@LC treatment successfully activates the STING signal pathway in tumors, induces M1 polarization of macrophages, promotes T cell infiltration and activation, and promotes the remodeling of the tumor immune microenvironment.

### 2.5 Inhibition of In Situ Breast Cancer and Lung Metastasis by HRB@LC

**FIGURE 6:**
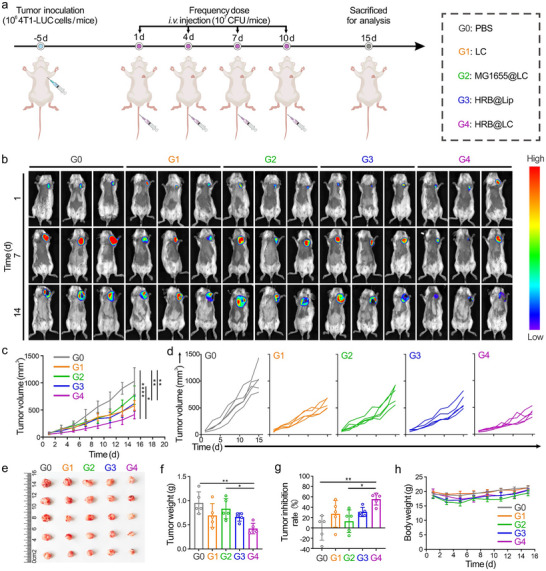
HRB@LC inhibits in situ tumor growth. (a) Schematic showing the treatment schedule in a situ breast cancer mouse model. (b) Bioluminescence images of mice, n = 3. (c) Tumor volume change curve of mice. Data are presented as the means ± SD (n = 5). (d) Tumor volume change curves of individual mouse, n = 5. (e) Tumor images obtained after different treatments, n = 5. (f) Tumor weights obtained after different treatments. Data are presented as the means ± SD (n = 5). (g) Tumor suppression rate obtained after different treatments. Data are presented as the means ± SD (n = 5). (h) Body weight change curve of mice. Data are presented as the means ± SD (n = 5). ^*^: *P* < 0.05. ^**^ *P* < 0.01. ^***^: *P* < 0.001.

We used 4T1‐LUC cells transfected with a luciferase reporter gene to establish an in situ breast cancer mouse model to further evaluate the in vivo antitumor effect of HRB@LC, following the treatment scheme shown in Figure 6a. We analyzed the antitumor effects of different treatment groups by detecting bioluminescence signals in tumor tissues of tumor‐bearing mice at a given time point. Figure 6b shows that at the start of treatment, there was almost no difference in the bioluminescence signals of the tumor areas among the five groups; however, in the middle and end of treatment, the bioluminescence signals of the G4 group were significantly lower than those of the other groups. The results of continuous measurement of tumor volume (Figure 6c,d) showed that the tumor volume was smallest in the G4 group, with significant differences compared to the other groups. On day 15, the tumors were collected for photography (Figure 6e) and weighing (Figure 6f). The results showed that the tumors in the G4 group were the smallest and lightest. In addition, the tumor inhibition rate in the G4 group reached 60% (Figure 6g), which was significantly higher than that in other groups. Figure 6h shows that there was no significant change in the body weight of mice during the entire treatment period. The above results collectively indicate that HRB@LC has good biosafety and can effectively inhibit the development of in situ breast cancer, showing significant antitumor activity.

**FIGURE 7:**
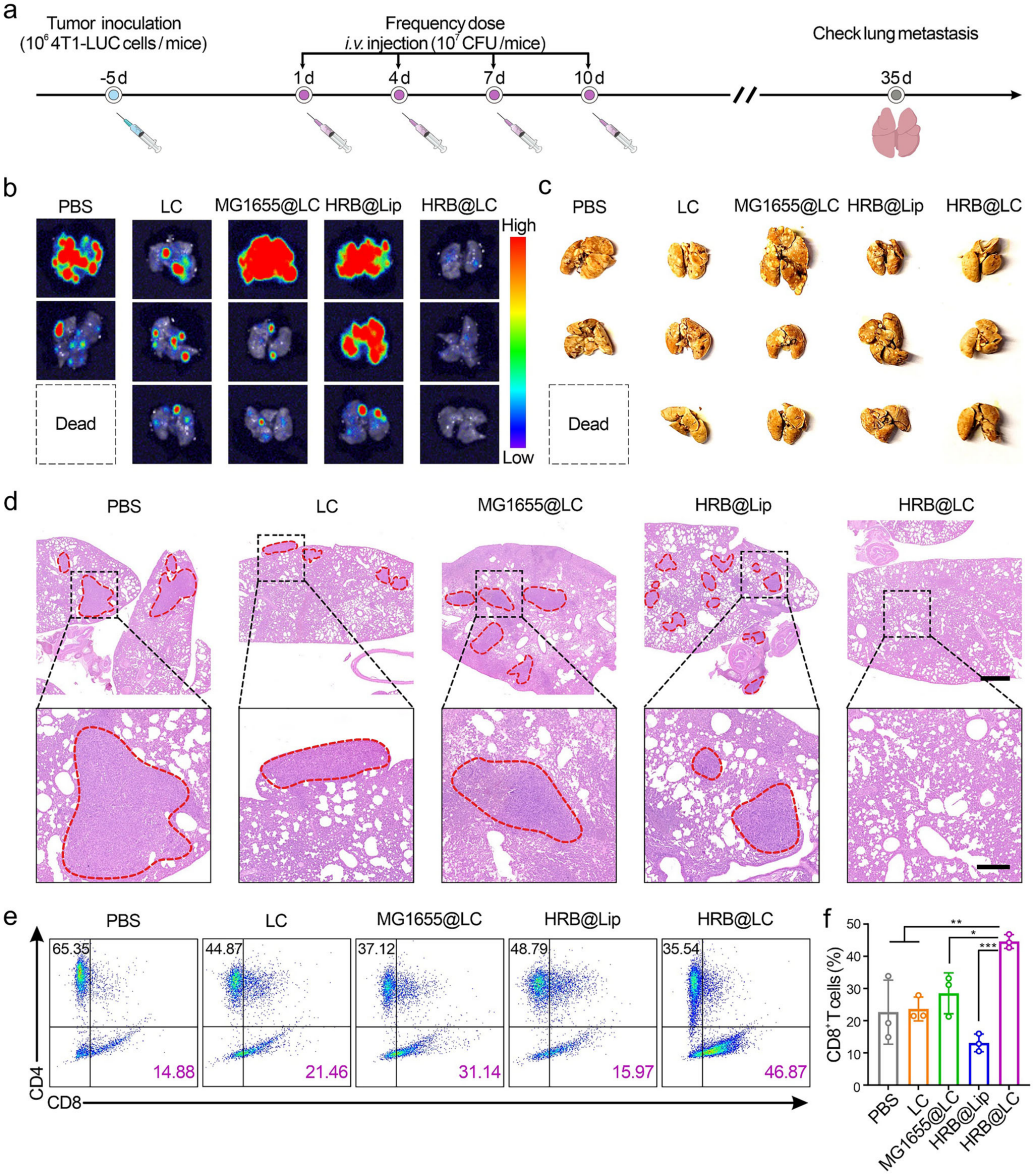
HRB@LC inhibits metastatic tumor. (a) Schematic illustration of the treatment schedule of metastatic tumor. (b‐c) Bioluminescence images of lung tissues, n = 3. (c) Lung tissues were fixed in Bouin's fixative for 48 h, n = 3. (d) Representative H&E staining images of lung tissues. Scale bars, 1 mm and 0.3 mm (enlarged view). Images were representative of three experiments. (e‐f) Representative flow cytometry (e) and quantitative analysis (f) of cytotoxic T cells (CD4^−^ CD8^+^) in spleen from mice after different treatments. Images were representative of three experiments. Data are presented as the means ± SD (n = 3). ^*^: *P* < 0.05. ^**^ *P* < 0.01. ^***^: *P* < 0.001.

To further evaluate the inhibitory effect of HRB@LC on tumor metastasis, we treated tumor‐bearing mice according to the experimental scheme shown in Figure 7a and monitored lung metastasis over the long term. Bioluminescence imaging was performed on the lung tissue of tumor‐bearing mice in each group, and the results are shown in Figure 7b. Except for the HRB@LC group, all other groups (PBS, MG1655@LC, HRB@Lip, and LC) showed obvious metastatic tumor cell signals, indicating the presence of lung metastases. Among them, the signal strength of the LC group was relatively low. After fixing lung tissue with Bouin's solution, we can see clear white metastatic nodules (Figure 7c). Almost no metastatic nodules were observed in the HRB@LC group, while all other groups had a large number of nodules. It is worth noting that the number of nodules in the LC group was significantly lower than that in the PBS, MG1655@LC, and HRB@Lip groups. H&E staining results (Figure 7d) further confirmed the above findings: compared with the large metastatic lesion areas in the lung tissues of the PBS, MG1655@LC, and HRB@Lip groups, the lesion areas in the LC group were smaller and fewer in number. More importantly, no metastatic lesion areas were observed in the lung tissues of the HRB@LC group, and its histological morphology was uniformly normal. The above results consistently indicate that HRB@LC can significantly inhibit lung metastasis of tumors, with LC playing a greater role in the long‐term inhibition of lung metastasis. To explore the potential mechanism, we analyzed T cells in mouse spleens using flow cytometry, and the results are shown in Figure 7e and Figure S15. The ratio of cytotoxic T cells (CD4^−^ CD8^+^) in the spleens of the HRB@LC group was significantly higher than that in other groups, reaching 45% (Figure 7f). These results suggest that HRB@LC may induce long‐term immune memory responses, which are closely associated with its successful suppression of metastatic tumor development.

## 3 Conclusion

We used synthetic biology and materials engineering methods to reprogram bacteria and construct the composite material HRB@LC, with the aim of overcoming the delivery limitations of ICIs such as aCD47 and achieving in situ antibody synthesis at the tumor site. STING agonists are crucial in activating the innate immune response of macrophages against tumors, but their high hydrophilicity and negative charge characteristics result in low cytoplasmic delivery efficiency and easy degradation and clearance. We used liposome encapsulation of STING agonists to effectively solve the above problems and achieve targeted delivery to the tumor site. After HRB@LC precisely targeted solid tumors, the hypoxic response expressed aCD47, which effectively inhibited immune escape. In addition, LC responds to the weakly acidic TME by releasing from the HRB surface and being taken up by macrophages, activating the STING signal pathway of macrophages and inducing IFN‐β expression, ultimately enhancing the systemic immune response. In subcutaneous and in situ breast cancer models in mice, HRB@LC shows significant antitumor effects and immune activation effects. At the same time, HRB@LC can stimulate long‐term immune memory and effectively inhibit the formation of metastatic tumors. In summary, HRB@LC combines excellent tumor targeting and biological safety, enabling in situ synthesis of ICIs at the tumor site and activation of the STING signal pathway, thereby generating a powerful anti‐tumor immune response. In terms of material preparation, we conducted repeated experiments to assess the particle size of LC, the encapsulation efficiency and drug loading of cGAMP, the loading amount of HRB on LC, as well as the efficacy in both cellular and mouse models. The results demonstrate good consistency and reproducibility in the preparation process and quality control of the material. HRB@LC represents a highly promising new strategy in the field of tumor immunotherapy. More importantly, beyond the demonstrated delivery of cGAMP and expression of aCD47, this platform can be further engineered to deliver other immune agonists or express different therapeutic antibodies. Using synthetic biology tools, modular plasmids encoding specific antibodies (such as aPD‐L1, aCTLA4, etc.) can be constructed and transfected into engineered bacteria for stable expression and release of the corresponding antibodies. Meanwhile, the hydrophilic core of liposomes is suitable for encapsulating hydrophilic molecules, while the phospholipid bilayer can be loaded with hydrophobic molecules. Previous studies [11, 37]. have shown that liposomes can effectively deliver Toll‐like receptor agonists, such as monophosphoryl lipid A, imiquimod, and poly(I:C). Therefore, we believe this “engineered bacterial factory” platform holds broad application prospects.

## 4 Experimental Section/Methods

### 4.1 Preparation of HRB

Clone the genes encoding the *fdhF* promoter, *aCD47*, and *mCherry* into the pBV220 plasmid to form a reprogrammable plasmid. Using reverse transcription technology, sequence the plasmid, confirm the sequence is correct, and then amplify and extract the plasmid. Take the competent *E. coli* MG1655 from ‐80°C and place it on ice. After thawing for 5 mins, add the prepared reprogrammable plasmid to a 1.5 mL EP tube and gently stir the bottom of the centrifuge tube to promote mixing. Place the 1.5 mL EP tube in a 42°C water bath for 45 s, then immediately place it on ice for 2 mins. Add 700 µL LB medium to the EP tube, place it in a shaking incubator at 37°C and 200 rpm, and incubate for 1 h. Take the EP tube out and centrifuge at 3500 rpm for 5 mins, collect the bacterial pellet, dilute it, and spread it on LB solid medium containing ampicillin (50 µg/mL). Incubate it overnight at 37°C. Pick a single colony from the LB solid medium and transfer it to LB medium. Incubate in a shaking incubator at 37°C and 220 rpm until the OD_600_ reaches 0.6‐1.0 (at this point, the bacteria are in the logarithmic growth phase) for subsequent experiments.

### 4.2 CD47 Antibody Expression by HRB

Pick a single colony of HRB from a solid LB agar plate containing ampicillin (50 µg/mL) and transfer it to LB medium. Incubate it in a shaking incubator at 37°C and 220 rpm. Collect bacterial cultures at different time points and obtain fluorescent images using the GelView6000proII fluorescent chemiluminescence imaging system. HRB was cultured in both normoxic and hypoxic environments, and HRB fluid was collected at different time points. The content of aCD47 in the supernatant was detected using a CD47 ELISA kit. Take MG1655 and HRB single colonies in LB medium and culture them separately in normal oxygen and hypoxic environments for 12 h. Collect the supernatant and cell pellet from the four bacterial cultures (the cell pellet was treated with RIPA lysis buffer to release proteins), and perform SDS‐PAGE experiments to detect aCD47 and mCherry in the supernatant and cell pellet of the four bacterial cultures.

### 4.3 Permeation of HRB@LC in 4T1 Cells Spheroids

Prepare 1% agarose in PBS, autoclave at 121°C for 20 mins, and spread it on the bottom of a 96‐well plate while still hot. Digest and centrifuge 4T1 cells in logarithmic growth phase, and seed 4T1 cells at a density of 2000–10000 cells/200 µL/well into the above‐mentioned treated 96‐well plate. After centrifugation at 1500 rpm for 10 mins, the cells formed clusters. After incubating them in a cell culture incubator for 48 h, the medium was replaced to obtain 4T1 cells spheroids. Incubate HRB@LC (10^7^ CFU/mL) with tumor spheroids for 6 h, wash three times with PBS, and stain with Hoechst 33342 for 10 mins, then wash three times with PBS. Observe using FV3000 confocal microscope and perform Z‐axis scanning imaging.

### 4.4 In Vivo Tumor Targeting of HRB@LC

In LC preparation steps (Supporting Information), add 25 µL of DiR dye at a concentration of 5 mg/mL under magnetic stirring in the dark. After hydration, centrifuge for 5 mins through a 3 kDa ultrafiltration tube, repeat twice, and resuspend the precipitate in PBS to obtain LC‐DiR. In vitro, the fluorescence intensity was detected by fluorescence imaging to roughly determine the content of LC‐DiR in HRB@LC‐DiR. HRB@LC‐DiR and an equal amount of LC‐DiR were injected into tumor‐bearing mice via the tail vein. At specified time points, in vivo fluorescence imaging was performed on mice to evaluate the distribution of HRB@LC‐DiR and LC‐DiR in vivo. After injection 24 h, tumor‐bearing mice were euthanized. Their hearts, livers, spleens, lungs, kidneys, and tumor tissues were collected for in vitro fluorescence imaging. After imaging, the organs and tumor tissues were ground into a homogenate using sterile water, spread on solid LB agar plates containing ampicillin (50 µg/mL), and incubated at 37°C overnight.

### 4.5 Activation of the STING Signal Pathway in Macrophages

Place HRB@LC in PBS solution at pH 6.5 and treat for 6 h. Centrifuge at 3500 rpm for 5 mins, then collect the supernatant. The control groups were the supernatant obtained after treating HRB@LC in PBS solution at pH 7.4 for 6 h, the supernatant obtained after treating HRB@Lip (without cGAMP) in PBS solution at pH 6.5/pH 7.4 for 6 h, and an equal amount of LC. Add the supernatant from each group to DMEM, slowly adjust the pH of the solution to 7.4 with low‐concentration NaOH, and incubate with RAW 264.7 cells for 4 h. Collect cell supernatants and use an IFN‐β ELISA test kit to detect the IFN‐β content. Cells were treated with RNA isolator and total RNA was obtained, referring to the instructions. Use the HiScript@III All‐in‐one RT SuperMix Perfect for qPCR Kit to reverse transcribe the total RNA samples into cDNA. RT‐qPCR was performed using the Taq Pro Universal SYBR qPCR Master Mix kit and the QuantStudio 6 Pro real‐time fluorescent quantitative PCR instrument. The sequences of primers for *Ifnb1, CCL‐5* genes were listed in Supplementary Information.

### 4.6 Analysis of Immune Checkpoint Blockade in Macrophages

Take MG1655 and HRB single colonies in LB medium and culture them separately in normal oxygen and hypoxic environments for 12 h. Collect the supernatants of the four bacterial cultures by centrifuging at 3500 rpm for 5 mins, filter them through a 0.22 µm filter membrane, and concentrate them using a 3 kDa ultrafiltration tube for future use. Seed 4T1 cells into a 6‐well plate (1.5 × 10^5^ cells/well) and incubate overnight. Add the supernatant mentioned earlier to DMEM at a ratio of 1:100 and incubate with 4T1 cells for 8 h. Collect 4T1 cells, add DiR dye, and incubate at 37°C for 30 mins to obtain DiR‐labeled 4T1 cells. Add DiR‐labeled 4T1 cells to RAW 264.7 cells (4.5 × 10^5^ cells/well) and incubate at 37°C for 18 h. Wash the cells three times with PBS, add F4/80 (Biolegend, Cat No. 123107, dilution ratio 1:100) to stain the RAW 264.7 cells, observe phagocytosis using the FV3000 confocal microscope, and the DiR^+^ cells in the RAW 264.7 cells were analyzed by flow cytometry.

### 4.7 In Vivo Antitumor Efficacy

The subcutaneous breast cancer model was established by injecting 1×10^6^ 4T1 cells into the right thigh of 6‐week‐old female Balb/c mice. The in situ breast cancer model was created by injecting 1×10^6^ 4T1‐LUC cells into the left upper mammary gland of 6‐week‐old female Balb/c mice. When the tumor volume reached 80–100 mm^3^, the mice were randomly divided into five groups (n = 5), including the PBS group (G0), LC group (G1), MG1655@LC group (G2), HRB@Lip group (G3), and HRB@LC group (G4). Among them, the dose for the G2‐G4 group was 10^7^ CFU, and the dose for the G1 group was 5 µL. Record the weight and tumor volume of mice every other day, and perform in vivo bioluminescence imaging on mice with an in situ breast cancer model at a given time point. Specifically, D‐fluorescein potassium salt is dissolved in PBS to prepare a 30 mg/mL solution, which is then thoroughly dissolved by ultrasonication and filtered through a 0.22 µm filter membrane to remove bacteria. Administer the D‐fluorescein potassium salt solution via intraperitoneal injection into mice 10–15 mins prior to in vivo imaging, at a dose of 3 mg per mouse (100 µL/mouse). Euthanize the mice at a specified time point (15 days or 21 days), and their tumor tissues and major organs (heart, liver, spleen, lungs, and kidneys) were collected. Photograph and weigh the tumor tissues, and organs are immersed in a 4% polyformaldehyde solution for 48 h for fixation for H&E staining.

To further analyze the immune response, the subcutaneous tumor‐bearing mice were euthanized 36 h after the first dose (n = 3). Their tumors, spleens, and lymph nodes (TDLN) were collected, washed with PBS, and ground to obtain a single‐cell suspension. Then the cells were stained with the following surface antibodies: F4/80 (Biolegend, Cat No. 123116, dilution ratio 1:100), CD86 (Biolegend, Cat No. 105006, dilution ratio 1:100), CD206 (Biolegend, Cat No. 141706, dilution ratio 1:100), CD80 (Biolegend, Cat No. 104708, dilution ratio 1:100), CD3 (Biolegend, Cat No. 100206, dilution ratio 1:100), CD8 (Biolegend, Cat No. 100706, dilution ratio 1:100), CD11c (Biolegend, Cat No. 117310, dilution ratio 1:100). According to the manufacturer's instructions. The stained cells were analyzed by flow cytometry, and the data were analyzed using FlowJo. In addition, the relative expression levels of IFN‐β and other related cytokines were detected in mouse tumor tissues through RT‐qPCR. Tumors were treated with RNA isolater and total RNA was obtained, referring to the instructions. Use the HiScript@III All‐in‐one RT SuperMix Perfect for qPCR Kit to reverse transcribe the total RNA samples into cDNA. RT‐qPCR was performed using the Taq Pro Universal SYBR qPCR Master Mix kit and the QuantStudio 6 Pro real‐time fluorescent quantitative PCR instrument. The sequences of primers for these genes were listed in Supplementary Information. The specific procedure for immunofluorescence sectioning is as follows: After the tumor tissues were collected, they were immersed in a 4% polyformaldehyde solution for 48 h for fixation, followed by immersion in a 30% sucrose solution for 24–36 h until the tumor tissues dehydrated and settles. Embed the tumor tissue using an embedding agent, and use the CM1950 low‐temperature cryostat to cut the tumor tissue into 4–8 mm thick tissue sections. Use the FITC Tunel Cell Apoptosis Detection Kit (Servicebio, China) to perform TUNEL detection on tumor sections. Use Ki67, CD8, CD86 antibodies and Cy3‐labeled goat anti‐rabbit IgG (Servicebio, Cat No. GB21303, dilution ratio 1:200) to stain tumor sections separately.

### 4.8 Ethics

All animal experiments were approved by the Animal Experiment Ethics Committee of Huazhong University of Science and Technology (IACUC Number: 4208).

### 4.9 Statistical Analysis

Data are expressed as mean ± S.D. Significance between two groups was assessed by an unpaired two‐tailed Student's t‐test, and between each of the multiple groups was calculated using one‐way ANOVA. Values with P < 0.05 were considered significant. *: P < 0.05. ^**^ *P* < 0.01. ^***^: *P* < 0.001. All the statistical analyses were performed using GraphPad Prism (8.0). Flow‐cytomery data were analyzed with FlowJo (ver. 10.8.1). Confocal images were analyzed with FluoView31S (ver.2.3.1.163).

Experimental details are described in the Supplementary Information.

## Conflicts of Interest

The authors declare no conflicts of interest.

## Supporting information




**Supporting File**: advs74460‐sup‐0001‐SuppMat.pdf

## Data Availability

The data that support the findings of this study are available from the corresponding author upon reasonable request.
