# Supplementary material for: Engineered Bacteria Factory Integrating Drug Delivery and Antibody Manufacture for Activating the STING Signal Pathway Mediated Tumor Immunotherapy
Source: Adv Sci (Weinh). 2026 Feb 19;13(27):e18172. doi: 10.1002/advs.202518172 (PMC13170238; doi:10.1002/advs.202518172)
Supplement: Supplementary file 1 — Supporting File: advs74460‐sup‐0001‐SuppMat.pdf [file ADVS-13-e18172-s001.pdf]

*Supporting Information*

*of*

# Engineered Bacteria Factory Integrating Drug Delivery and Antibody Manufacture for Activating the STING Signal Pathway Mediated Tumor Immunotherapy

*Peng-Shuo Dong, Tian Yao, Yong Li, Zi-Hui Yan, Xiao-Ting Xie, Jia-Hua Zou, Kai Cheng*, Jin-Xuan Fan*, Yuan-Di Zhao*, Qiu-Ran Xu**

Peng-Shuo Dong, Tian Yao, Zi-Hui Yan, Dr. Xiao-Ting Xie, Jia-Hua Zou, Dr. Kai Cheng, Dr. Jin-Xuan Fan, Prof. Yuan-Di Zhao
Britton Chance Center for Biomedical Photonics at Wuhan National Laboratory for Optoelectronics - Hubei Bioinformatics & Molecular Imaging Key Laboratory, Department of Biomedical Engineering, College of Life Science and Technology, Huazhong University of Science and Technology, Wuhan 430074, Hubei, P. R. China

Prof. Qiu-Ran Xu
Zhejiang Key Laboratory of Tumor Molecular Diagnosis and Individualized Medicine, Zhejiang Provincial People's Hospital Affiliated People's Hospital, Hangzhou Medical College, Hangzhou 310014, Zhejiang, P. R. China

Jia-Hua Zou
Department of Oncology, Huanggang Central Hospital of Yangtze University, Huanggang 438000, Hubei, P.R. China

Jia-Hua Zou
Hubei Clinical Medical Research Center of Esophageal and Gastric Malignancy, Huanggang 438021, Hubei, P.R. China

Dr. Kai Cheng
Department of Chemistry, The Chinese University of Hong Kong, Hong Kong SAR 99999, P.R. China

E-mail: kaicheng@cuhk.edu.hk (K. Cheng), jxfan@hust.edu.cn (J.X. Fan), zydi@mail.hust.edu.cn (Y.D. Zhao), xuqiuran@hmc.edu.cn (Q.R. Xu)

**Methods**

**Materials**

The aCD47 peptide gene sequence was obtained from NCBI and synthesized by Miaoling Bioscience & Technology Co. (Wuhan, China). Lecithin, stearic acid and cGAMP were purchased from Shanghai Aladdin Biochemical Technology Co., Ltd. (Shanghai, China). DSPE-PEG-CHO M.W.2000, DSPE-PEG M.W.2000 and DSPE-FITC were purchased from GuangZhou Tanshtech Co.,Ltd. (GuangZhou , China). DiR was purchased from Shanghai Macklin Biochemical Co., Ltd. (Shanghai, China). CD47 enzyme-linked immunosorbent assay (ELISA) kits and IFN- β enzyme-linked immunosorbent assay (ELISA) kits were purchased from Bioswap Life Science Lab (Wuhan, China). Fetal bovine serum (FBS) was purchased from Zhejiang Tianhang Biotechnology Co., Ltd. (Zhejiang, China). Trypsin, PBS, and DMEM were purchased from Servicebio Biotechnology Co., Ltd. (Wuhan, China). RNA isolation reagent, HiScript III All-in-one RT SuperMix Perfect for qPCR kit, Taq Pro Universal SYBR qPCR Master Mix kit and One-Step PAGE Gel Fast Preparation Kit (15%) were purchased from Vazyme Biotech Co., Ltd. (Nanjing, China). DAPI, BCA Protein Assay Kit, penicillin-streptomycin, RNase-free dd-$H_2O$ and 4% paraformaldehyde fixative were obtained from Beyotime biotechnology Co.,Ltd. (Shanghai, China). Protein extraction kit was purchased from Bioswamp Life Science Lab (Wuhan, China). MTT (3-(4,5 Dimethylthiazol-2-yl)-2,5-diphenyltetrazolium bromide) was purchased from Sigma-Aldrich. Dimethyl sulfoxide (DMSO), sodium dodecyl sulfate (SDS), sodium hydroxide and hydrochloric acid were purchased from Sinopharm Chemical Reagent Co., Ltd. (Shanghai, China). Phenylmethanesulfonyl fluoride (PMSF), Tris (hydroxymethyl) aminomethane (Tris) and glycine were purchased from Beijing Kehbio Techology Co., Ltd. (Beijing, China). D-Luciferin, Potassium Salt was purchased from Shanghai Yingxin Laboratory Equipment Co., Ltd. (Shanghai, China). FITC Tunel Cell Apoptosis Detection Kit was purchased from Servicebio Biotechnology Co., Ltd. (Wuhan, China).

**Cell lines**

Mouse breast cancer cells (4T1) and mouse breast cancer cells-luciferase labelled (4T1-LUC) was obtained from Wuhan Hualianke Biotechnology Co., Ltd. Mouse mononuclear macrophage leukemia cells (RAW 264.7) was obtained from Wuhan Saitekang Biotechnology Co., Ltd. All cells were cultured in DMEM medium containing 10% fetal bovine serum, 100 U/mL penicillin and 0.1 mg/mL streptomycin.

**Animals**

Female BALB/c mice weighing 18 to 20 g (6-8 weeks old) were purchased from Beijing Weirhe Experimental Animal Technology Co., Ltd. Mice were housed in an animal facility under constant environmental conditions (room temperature, 22±1 °C ; relative humidity, 40-70%; and 12 hours light-dark cycle), and all mice had free access to food and water. Tumor volume was calculated as 0.5 × (length × width × width). To minimize animal discomfort, according to the Guideline of Assessment for Humane Endpoints in Animal Experiment (Certification and Accreditation Administration of the P. R. China, RB/T 173 2018), in general experiments, the tumor burden should not exceed 5% of the animal's normal body weight; in therapeutic experiments, it should not exceed 10% of the animal's body weight (10% indicated that the diameter of the subcutaneous tumor on the back of a 25 g mouse reached 17 mm). At the end of the mouse experiments, mice were euthanized according to animal welfare standards (euthanasia of all animals was performed using isoflurane in small animal anaesthetics). All animal experiments were approved by the Animal Experiment Ethics Committee of Huazhong University of Science and Technology (IACUC Number: 4208).

**Preparation of LC and HRB@LC**

Accurately weigh stearic acid (5 mg), lecithin (2 mg), and DSPE-PEG-CHO (3 mg), dissolve them in 1 mL of chloroform, stir magnetically for 10 mins while maintaining a stable liquid surface, connect to a vacuum pump for drying, and a white film will form at the bottom of the bottle. Add 200 μL of a 250 μg/mL cGAMP solution to the bottom of the bottle and sonicate

for 30 mins until the solution is clear. Collect the solution, centrifuge for 5 mins using a 30 kDa molecular weight ultrafiltration tube, repeat twice, resuspend the precipitate to obtain LC. Dilute LC 100 times and measure the hydrated particle size of LC. Drip LC onto a copper mesh, stain with 2% phosphotungstic acid, and observe the morphology of LC using a transmission electron microscope. The UV/Vis absorption spectrum shows that cGAMP has a characteristic ultraviolet absorption peak at 256 nm. Use an ultra-micro spectrophotometer to detect UV/Vis absorption spectrum of feed solution during LC preparation (250 μg/mL cGAMP solution), and filtrate obtained after LC ultrafiltration. Based on the absorption peak value at 256 nm, the cGAMP concentration and content were calculated. Use the formula

$$EE\ (\%) = (WA\text{-}WB)/WA \times 100\%$$

to calculate the drug encapsulation rate, where WA is the drug content at the time of feeding and WB is the drug content in the filtrate.

Collect HRB solution in the logarithmic growth phase, centrifuge at 3500 rpm for 5 mins, and wash the bacterial pellet three times with PBS. Then add HRB and LC in proportion to the bottom of a 10 mL EP tube and stir overnight at 4 °C. Centrifuge at 3500 rpm for 5 mins, remove the supernatant, wash the pellet three times, and HRB@LC is obtained. Preparation of LC and HRB@LC were performed in physiological-condition simulated PBS.

**Cytotoxicity of HRB@LC**

Prepare HRB@LC and add it to DMEM to obtain HRB@LC culture medium at different concentrations. Incubate with 4T1 cells seeded in a 96-well plate at 37 °C for 4 hours. Wash the cells three times with PBS. Add 20 µL of 5 mg/mL MTT solution (freshly prepared) to each well and continue incubating at 37 °C in the dark for 4 hours. Remove the MTT solution, add 200 µL of DMSO solution to each well, and incubate at room temperature in the dark on a horizontal shaking incubator for 30 mins. Measure the absorbance at 490 nm using a full-wavelength microplate reader.

**Phagocytose of LC by macrophages**

Prepare HRB@LC-FITC, treat with a pH 6.5 solution, centrifuge at 3500 rpm for 5 mins to collect the supernatant, slowly adjust the solution pH to 7.4 with low-concentration NaOH, and incubate with RAW 264.7 cells. Collect cells at different time points, wash three times with PBS, fix cells with 4% polyformaldehyde , add DAPI for staining for 10 mins, wash three times with PBS. Then we observed phagocytosis using the FV3000 confocal microscope, and the FITC content in RAW 264.7 cells were analyzed by flow cytometry.

**Inhibittion of metastatic tumor by HRB@LC**

For lung metastasis treatment, treated mice (n = 3) were injected with D-fluorescein potassium salt solution via intraperitoneal injection on day 35. Euthanasia was performed 10–15 mins after injection, and the lungs and spleens of the mice were immediately collected. Bioluminescence imaging was performed on the lungs, and their weights were recorded. The lungs were then immersed in Bouin’s fixative for 48 hours, photographed to visualize white metastatic nodules, and stained with H&E for a comprehensive analysis of metastatic spread. The spleen was washed with PBS, ground to obtain a single-cell suspension. Then the cells were stained with the following surface antibodiesand: CD3 (Biolegend, Cat No. 100206, dilution ratio 1:100), CD8 (Biolegend, Cat No. 100706, dilution ratio 1:100) CD4 (Biolegend, Cat No. 100516, dilution ratio 1:100) According to the manufacturer’s instructions. The stained cells were analyzed by flow cytometry.

**Isolation and Culture of BMDM**

Bone marrow-derived macrophages (BMDM) were isolated from 6-week-old BALB/c mice. After euthanasia by cervical dislocation, the mice were soaked in alcohol for 5 mins. Under sterile conditions in a biological safety cabinet, the tibias and femurs were aseptically dissected and then soaked in alcohol for 3 mins. Pre-cooled PBS was drawn into a syringe and used to flush the marrow cavity repeatedly from one end of each bone until the bone turned white. The collected bone marrow cells were filtered through a 70 μm sterile cell strainer and centrifuged

at 1000 rpm for 5 mins. The pellet was resuspended in red blood cell lysis buffer to remove erythrocytes. After centrifugation, the cells were resuspended in complete DMEM medium supplemented with 20 ng/mL M-CSF and seeded into culture dishes. Cells were cultured for 7 days at 37°C under 5% $CO_2$, with the medium replaced every 3 days. On day 7, the adherent cells were collected as BMDM for subsequent experiments.

**The primer sequences used in the RT-qPCR experiment are shown below:**

Ifnb1 (IFN-β) Forward: GCGTTCCTGCTGTGCTTCTCC

Ifnb1 (IFN-β) Reverse: TGAAGTCCGCCCTGTAGGTGAG

CCL-5 Forward: GCTGCTTTGCCTACCTCTCC

CCL-5 Reverse: TCGAGTGACAAACACGACTGC

IL-6 Forward: CTTCCAGCCAGTTGCCTTCTTG

IL-6 Reverse: TGGTCTGTTGTGGGTGGTATCC

TNF-α Forward: ATGTCTCAGCCTCTTCTCATTC

TNF-α Reverse: GCTTGTCACTCGAATTTTGAGA

IFN-γ Forward: GACGCTTATGTTGTTGCTGATGGC

IFN-γ Reverse: CTGGAGGAACTGGCAAAAGGATGG

GAPDH Forward: GGCAAATTCAACGGCACAGTCAAG

GAPDH Reverse: TCGCTCCTGGAAGATGGTGATGG

**Supplementary Figures**

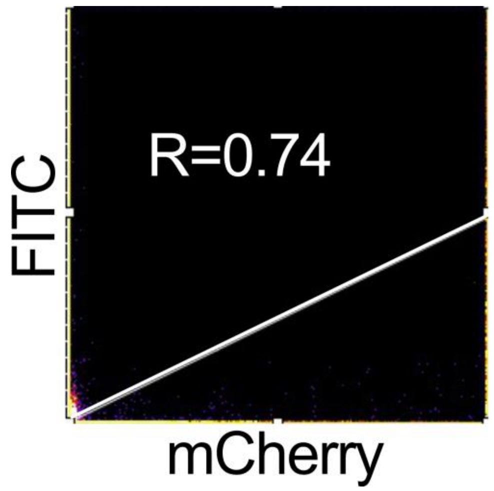


**Figure S1.** Colocalization analysis of mCherry and FITC in HRB@LC (Figure 2k). Each dot represents a pixel, with its coordinates defined by its intensity in the mCherry and FITC channels. Analysis was performed using ImageJ, yielding a Pearson's correlation coefficient (R) of 0.74.

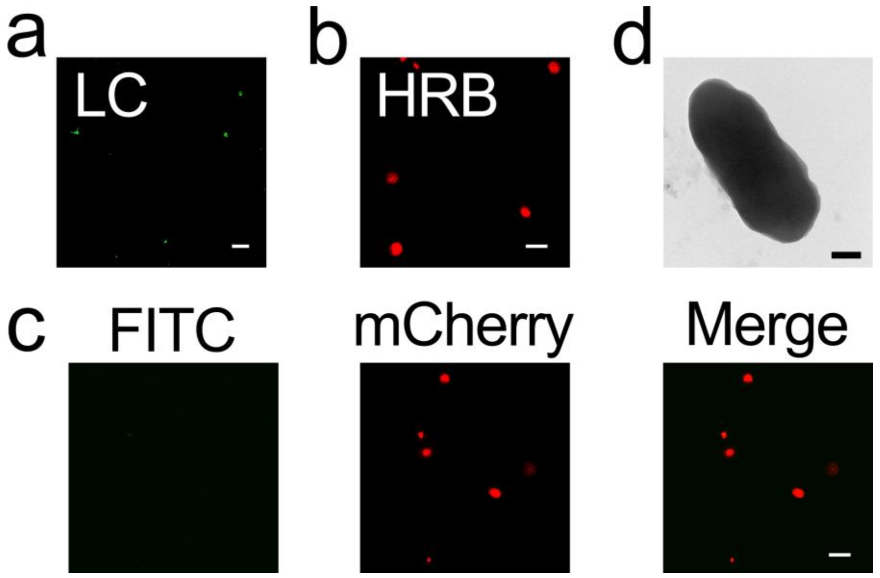


**Figure S2.** Confocal microscopy images of LC-FITC (without -CHO) (a) and HRB (b). Images were representative of three experiments. Scale bar, 5 µm (LC) and 10 µm (HRB). (c) Confocal microscopy images of HRB@LC (without -CHO). Images were representative of three experiments. Scale bar, 10 µm. (d) TEM images of HRB@LC (without -CHO). Scale bars, 250 nm.

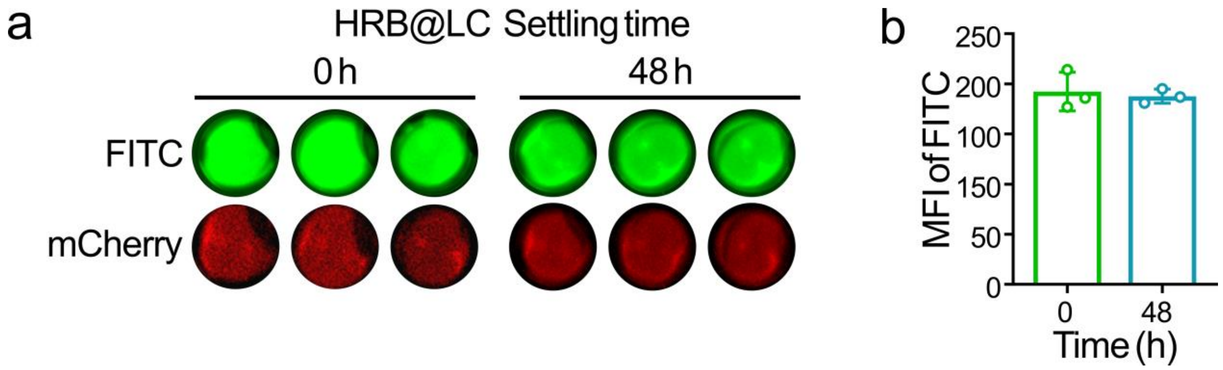


**Figure S3.** (a) Fluorescence images of HRB@LC. (b) Quantitative analysis of LC-FITC in HRB@LC. Data are presented as the means ± SD (n = 3).

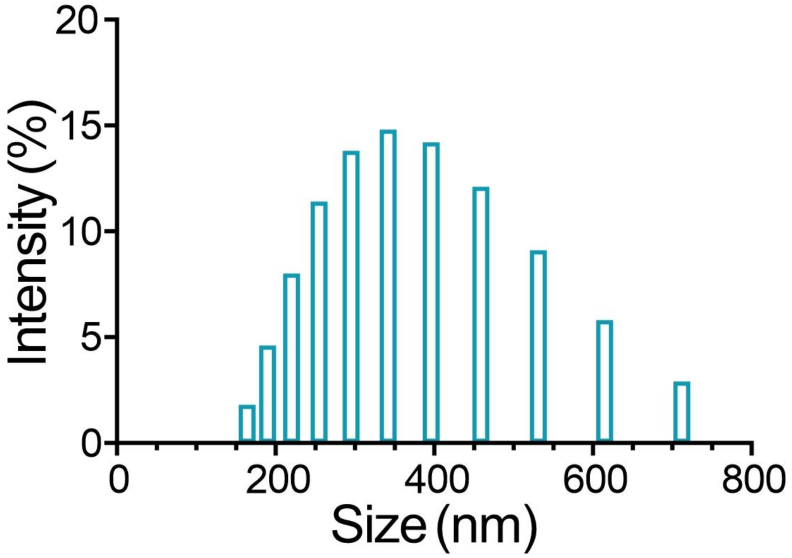


**Figure S4.** Hydrated particle size distribution graph of the supernatant obtained after a weak acidic environment of HRB@LC.

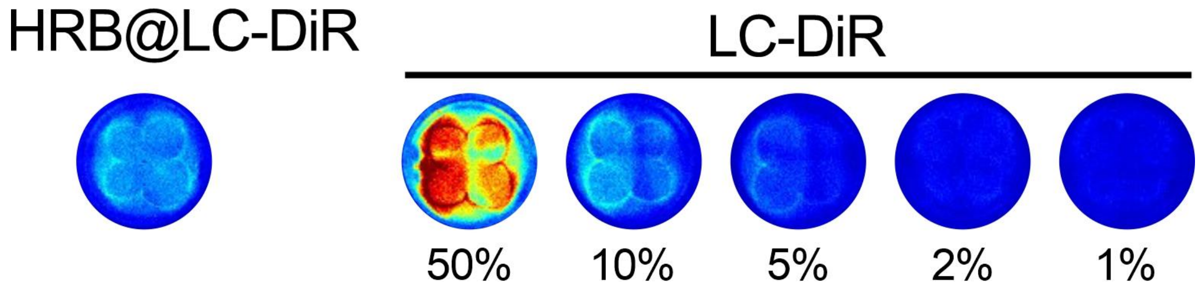


**Figure S5.** Fluorescence images of HRB@LC-DiR and LC-DiR. Images were representative of three experiments.

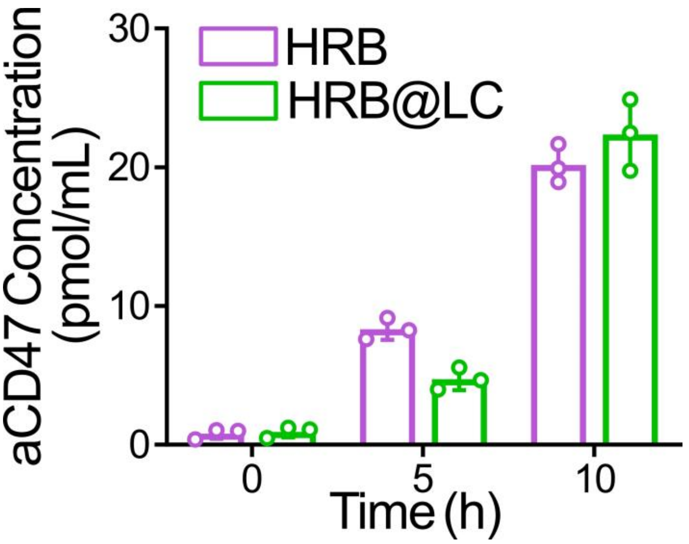


**Figure S6.** Quantitative analysis of aCD47 protein content in HRB@LC and HRB supernatant. Data are presented as the means ± SD (n = 3).

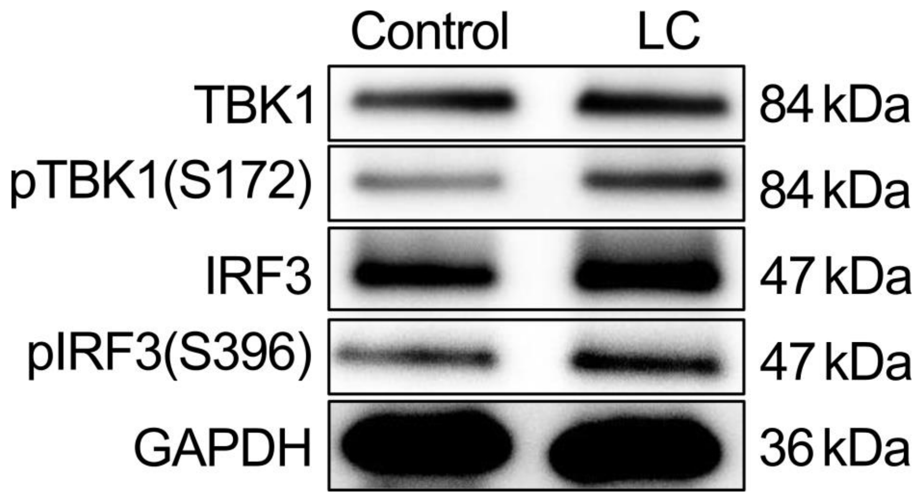


**Figure S7.** Western blot detection of STING pathway activation in BMDM after treated with LC for 10 h.

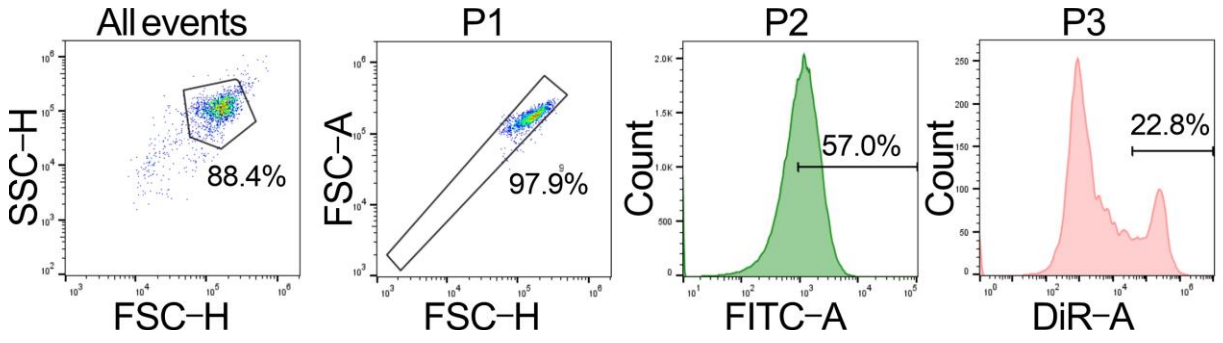


**Figure S8.** Gating strategy of Figure 4n.

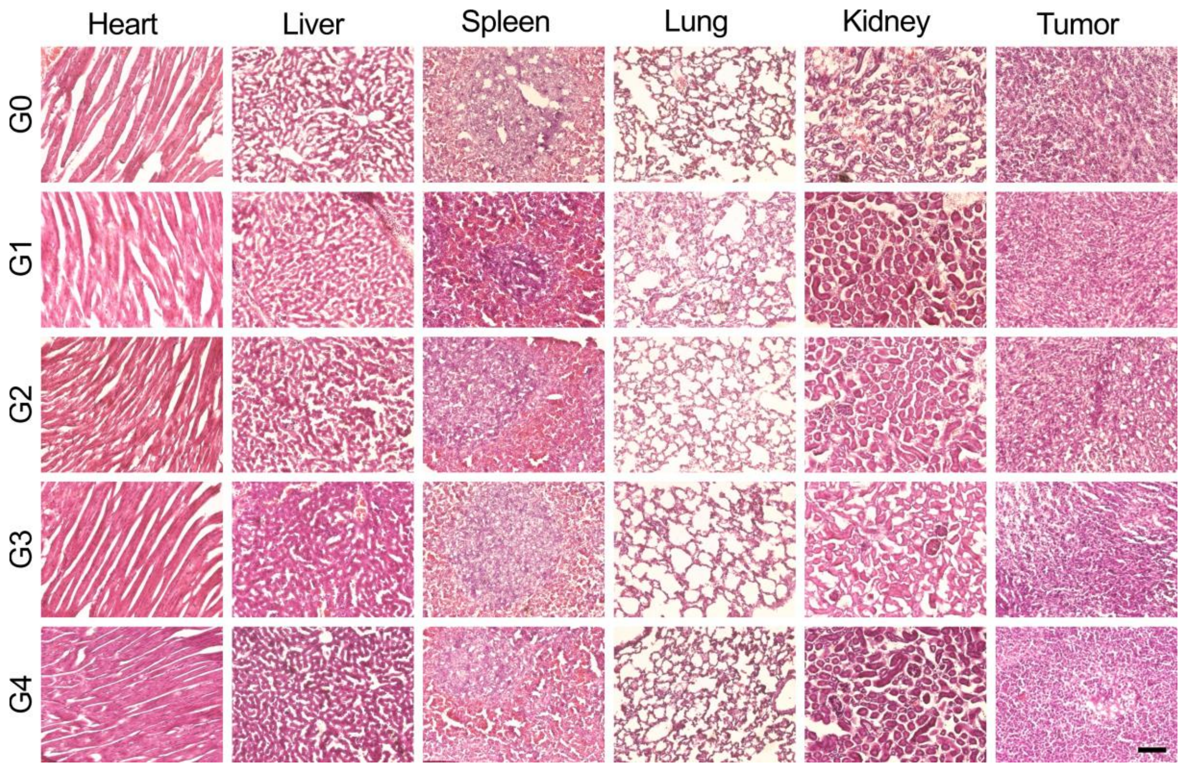


**Figure S9.** Representative H&E staining images of major organs and tumor tissues in mice. Scale bars, 0.1mm. Images were representative of three experiments.

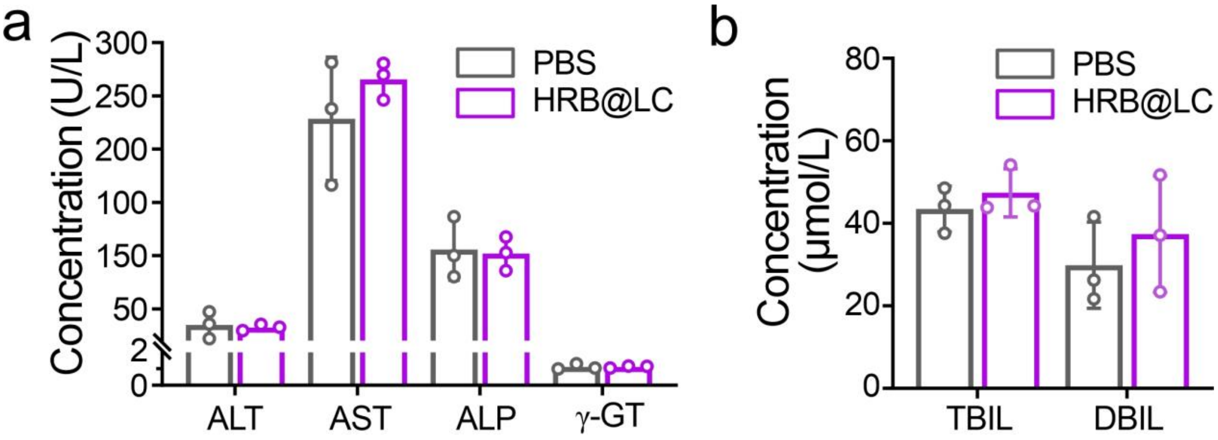


**Figure S10.** Serum liver function indicators from mice after different treatments. ALT represents Alanine Aminotransferase, AST represents Aspartate Aminotransferase, ALP represents Alkaline Phosphatase, γ-GT represents γ-Glutamyl Transferase, TBIL represents Total Bilirubin, DBIL represents Direct Bilirubin. Data are presented as the means ± SD (n = 3).

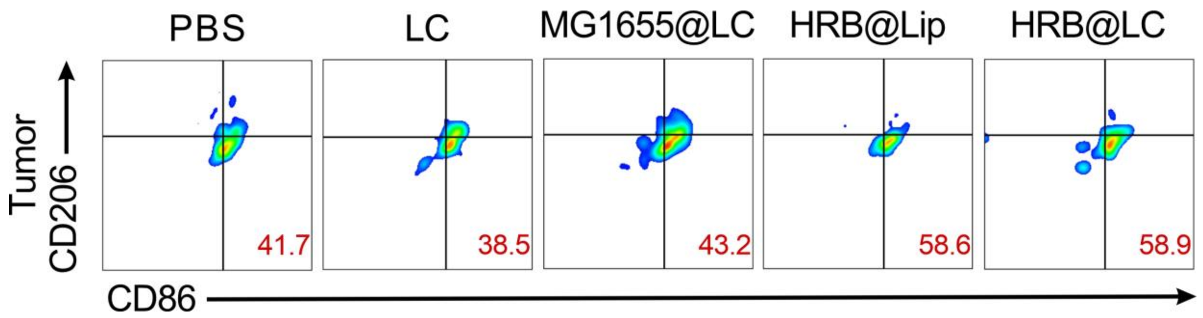


**Figure S11.** Representative flow cytometry of M1 macrophages ($CD86^+$ $CD206^-$) in tumor from mice after different treatments.

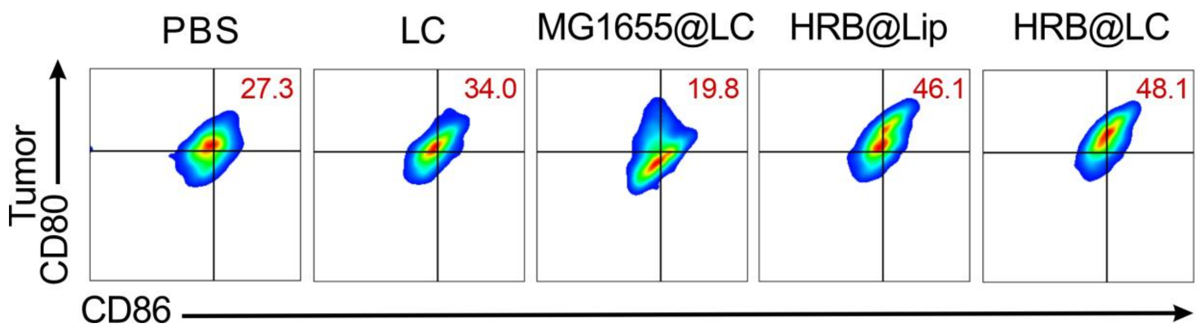


**Figure S12.** Representative flow cytometry of mature DCs ($CD80^+$ $CD86^+$) in tumor from mice after different treatments.

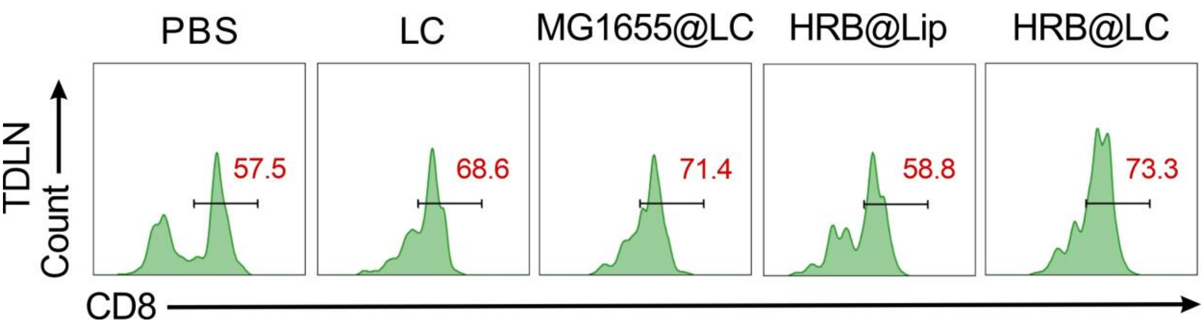


**Figure S13.** Representative flow cytometry of cytotoxic T cells ($CD3^+$ $CD8^+$) in TDLN from mice after different treatments.

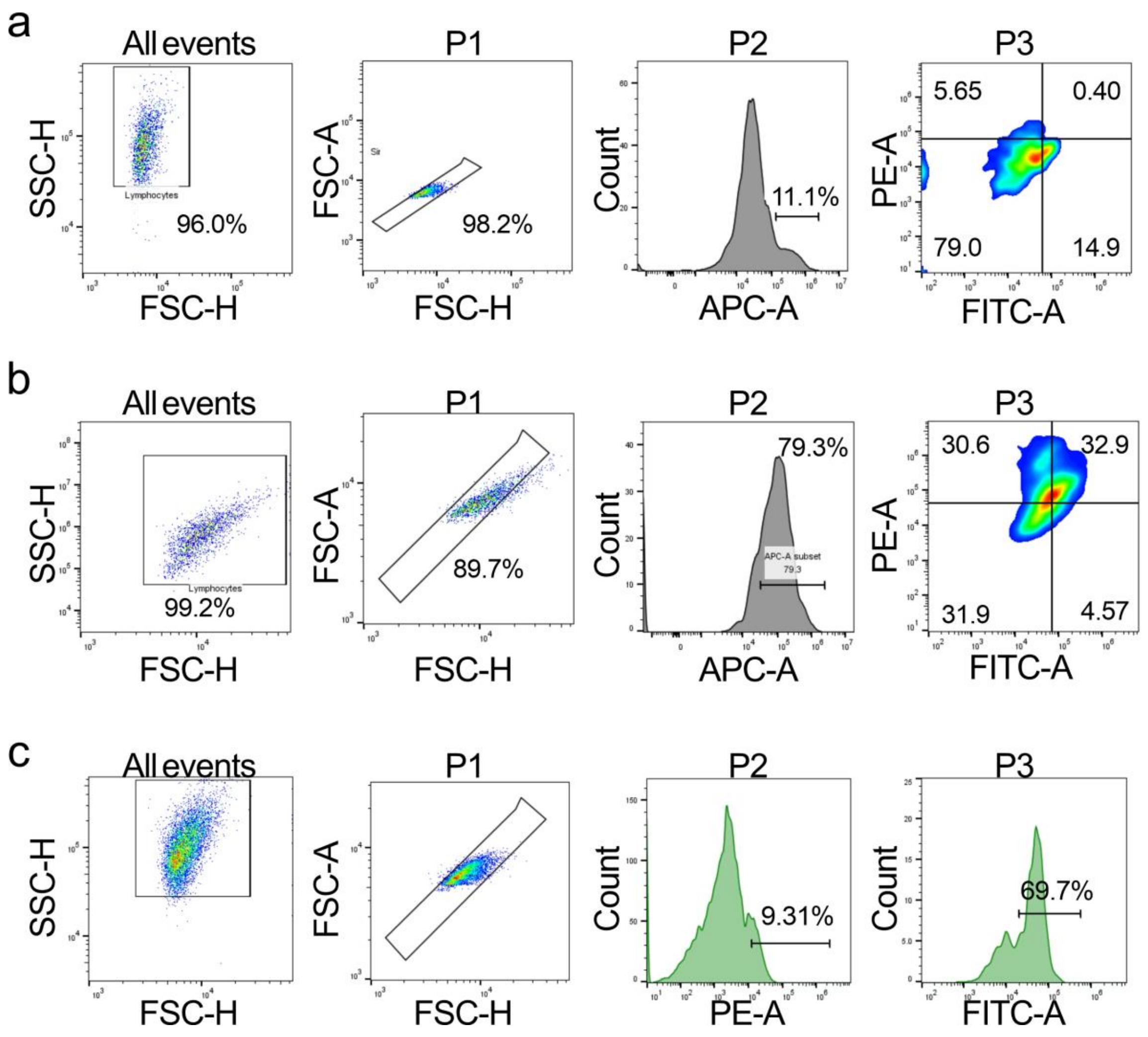


**Figure S14.** Gating strategy of Figure 5j (a),5k (b) and 5l (c).

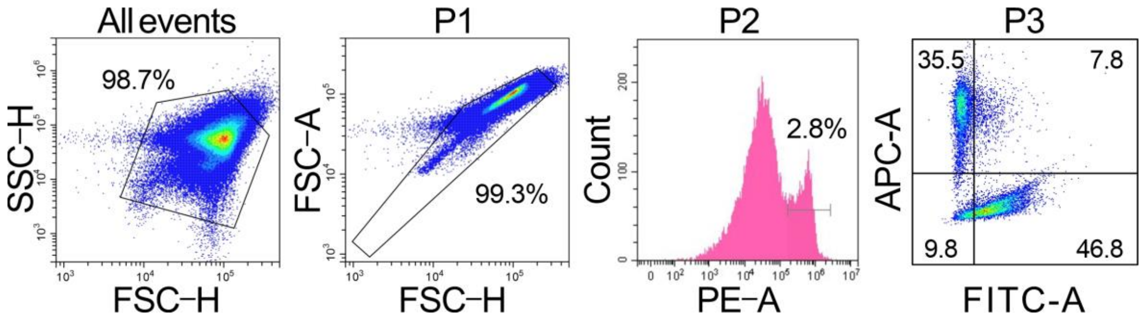


**Figure S15.** Gating strategy of Figure 7e.
